# Pin1 inhibition improves the efficacy of ralaniten compounds that bind to the N-terminal domain of androgen receptor

**DOI:** 10.1038/s42003-021-01927-3

**Published:** 2021-03-22

**Authors:** Jacky K. Leung, Yusuke Imamura, Minoru Kato, Jun Wang, Nasrin R. Mawji, Marianne D. Sadar

**Affiliations:** Department of Genome Sciences Centre, BC Cancer, Vancouver, BC Canada

**Keywords:** Cancer, Molecular biology, Prostate cancer, Hormone receptors

## Abstract

Therapies for lethal castration-resistant prostate cancer (CRPC) are an unmet medical need. One mechanism underlying CRPC and resistance to hormonal therapies is the expression of constitutively active splice variant(s) of androgen receptor (AR-Vs) that lack its C-terminus ligand-binding domain. Transcriptional activities of AR-Vs and full-length AR reside in its N-terminal domain (NTD). Ralaniten is the only drug proven to bind AR NTD, and it showed promise of efficacy in Phase 1 trials. The peptidyl-prolyl isomerase Pin1 is frequently overexpressed in prostate cancer. Here we show that Pin1 interacted with AR NTD. The inhibition of Pin1 expression or its activity selectively reduced the transcriptional activities of full-length AR and AR-V7. Combination of Pin1 inhibitor with ralaniten promoted cell cycle arrest and had improved antitumor activity against CRPC xenografts in vivo compared to individual monotherapies. These findings support the rationale for therapy that combines a Pin1 inhibitor with ralaniten for treating CRPC.

## Abstract (web summary)

Leung et al. find that the peptidyl-prolyl isomerase Pin1 targets the intrinsically disordered N-terminal domain of the androgen receptor (AR). They show that combining Pin1 inhibition with ralaniten compounds that bind to the AR N-terminal domain has enhanced antitumor activity on castration-resistant prostate cancer in xenografts, suggesting therapeutic potential.

## Introduction

Globally, there were 1.3 million new cases of prostate cancer in 2018. Approximately 30% of these patients will develop recurrence after primary treatment and require hormonal therapies. Currently, all FDA-approved hormonal therapies such as abiraterone acetate and enzalutamide target the C-terminal ligand-binding domain (LBD) of full-length androgen receptor (AR). About 20–40% of castration-resistant prostate cancer (CRPC) patients have de novo resistance to these agents, and virtually all patients will acquire resistance to AR-LBD-targeted therapy within 2–3 years^1^. Most CRPC continues to depend on AR transcriptional activity to maintain tumor growth and many involve the expression of constitutively active AR splice variants (AR-Vs) that lack the LBD. The most clinically relevant AR-V is AR-V7, which is detected in one-third of CRPC patients, associated with bone metastases, and resistance to abiraterone and enzalutamide^2,3^. Common to both full-length AR and AR-Vs is the N-terminal domain (NTD) that contains all of the AR’s transcriptional activity^4,5^. Thus, identifying drugs that disrupt the AR NTD should block the transcriptional activities of both full-length AR and AR-Vs. One hurdle in finding these drugs is the intrinsic disorder of this domain.

The first and currently only drug to be tested in clinical trials that directly binds to an intrinsically disordered protein is ralaniten^6^. Ralaniten binds to Tau-5 in AR NTD^7^ and inhibits the transcriptional activities of full-length AR and AR-Vs^8,9^. Proof-of-concept validation for the AR NTD as a drug target and the ralaniten scaffold for developing drugs to treat CRPC was provided by the first-in-human clinical trial (NCT02606123). A second-generation ralaniten analog (EPI-7386) is currently in Phase I clinical trial (NCT04421222).

To identify approaches that would disrupt the transcriptional activity of AR NTD, we considered the peptidyl-prolyl *cis/trans* isomerase Pin1. This enzyme is commonly overexpressed in prostate cancer, and its expression in patient biopsies correlates with recurrence^10–12^. Pin1 regulates the conformation of proteins by catalyzing the isomerization of proline bonds at specific phosphorylated motifs (pSer/Thr-Pro, Pin1 motif)^13,14^. Numerous putative binding sites for Pin1 are found within the intrinsically disordered AR NTD. Pin1 is composed of a WW domain that recognizes phosphorylated substrates joined by a flexible linker to a peptidyl-prolyl isomerase (PPIase) domain to carry out its catalytic function^15,16^. In the absence of Pin1, the rate of isomerization between the *cis-* and *trans* proline is inherently slow since bond rotation is impeded by the adjacent phosphoryl group^13,17^. Proline-directed Ser/Thr kinases (CDKs and MAPKs) and phosphatases (PP2A) that recognize and modify Pin1 sites are specific to the *trans-*proline conformation^18–20^. Thus, Pin1 is the only human isomerase that recognizes a proline-directed phosphorylation site, and Pin1-mediated *cis*/*trans* isomerization is proposed as a conformational switch for signal transduction^21^.

There is interest in Pin1 as a potential prognostic marker for prostate cancer, but there are no reports on whether Pin1 expression in prostate cancer influences AR signaling. Here we determine whether targeting Pin1 would be beneficial for treating CRPC. We investigated the mechanism and effect of genetic and pharmacological inhibition of Pin1 activity in CRPC and assessed the feasibility of combining a Pin1 inhibitor with ralaniten compounds using in vitro and in vivo models of CRPC.

## Results

### Pin1 is essential for the transcriptional activity of full-length AR

**Fig. 1:**
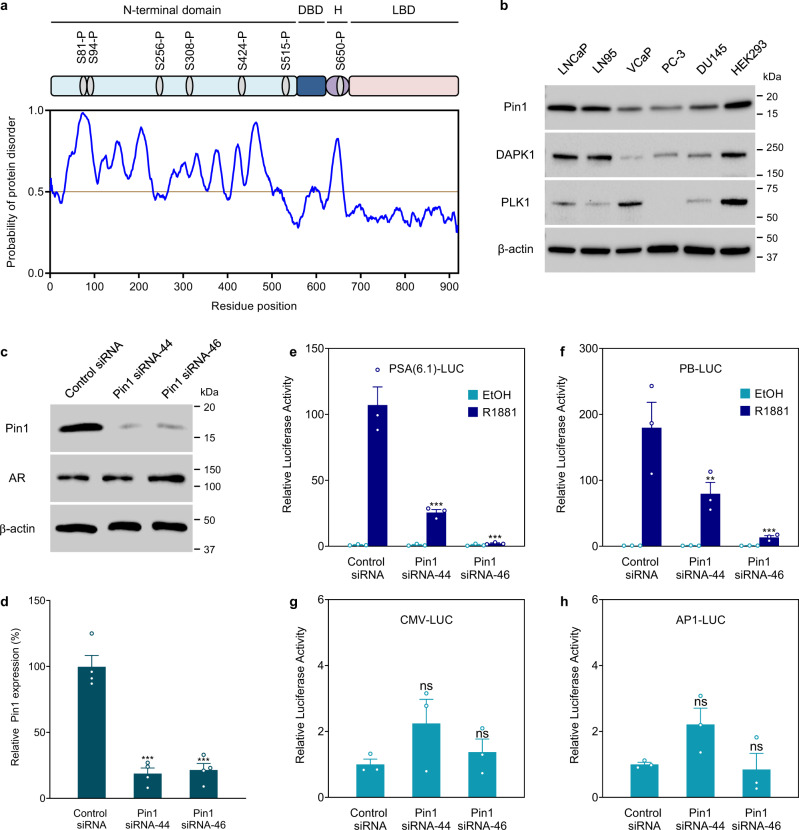
Pin1 is essential for the ligand-dependent transcriptional activity of AR. **a** Schematic depicting the position of putative Pin1 motifs on AR, which are based on experimentally verified phosphorylation sites from the Phospho.ELM database. Below, the RONN plot shows regions of predicted protein disorder where scores are above the 0.5 threshold. The numbering of residues is based on the 919 amino acid reference sequence for human AR, NCBI Accession No. AAA51729.1. DBD DNA-binding domain, H hinge region, LBD ligand-binding domain. **b** Western blot analysis of Pin1, DAPK1, and PLK1 expression in human prostate cancer cell lines and HEK293 cells (positive control) maintained in media supplemented with serum. **c** Pin1 protein levels in LNCaP cells after incubating with siRNA for 48 h. A non-targeting siRNA was used as a control. **d** Graph summarizing the knockdown efficiencies of the Pin1 siRNAs. **e**, **f** The activities of AR-driven reporters PSA(6.1 kb) and Probasin (PB) luciferase in LNCaP cells incubated with siRNA and androgen (R1881, 1 nM) for 48 h. **g**, **h** Activities of CMV- and AP-1-luciferase reporters which are not regulated by AR. Results shown are the means ± s.e.m. from three independent experiments. Statistical significance was determined by two-way ANOVA using Dunnett’s multiple comparisons test. **P < 0.01, ***P < 0.001; ns, not significant.

The AR NTD contains several putative binding sites for Pin1 (Fig. 1a). The levels of Pin1 protein were detected in all five of the human prostate cancer cell lines examined and were highest in androgen-sensitive LNCaP cells and lower in AR-negative PC-3 and DU145 cells (Fig. 1b). In general, Pin1 protein expression was approximately threefold higher in cell lines that express AR (LNCaP, LN95, and VCaP) than those that do not rely on AR for growth and survival (PC-3 and DU145; Supplementary Fig. 1a). In clinical samples of CRPC, transcript levels of PIN1 had a positive correlation with AR expression (Supplementary Fig. 1b). Analysis of expression of several transcriptional targets of AR in CRPC revealed positive correlations between the levels of PIN1 and KLK3 (PSA), TMPRSS2, and NKX3.1, but not FKBP5 (Supplementary Fig. 1c–f). These results were consistent with clinical data of elevated expression of Pin1 levels in patient biopsies that are associated with an increased likelihood of developing PSA recurrence after radical prostatectomy compared to patients with low expression of Pin1^12^.

Although levels of PIN1 mRNA were elevated in clinical samples of neuroendocrine prostate cancer that have low or no expression of AR (Supplementary Fig. 1g, h)^22^, there was no association between levels of PIN1 and AR (Supplementary Fig. 1i). Thus, the lower expression of Pin1 protein detected in DU145 and PC-3 cells may be a cell line-specific phenomenon. These clinical data show that Pin1 was expressed in both CRPC and neuroendocrine prostate cancer, and importantly that levels of PIN1 mRNA in CRPC were associated with expression of AR and its downstream transcriptional targets.

The catalytic activity of Pin1 can be regulated by phosphorylation by death-associated protein kinase 1 (DAPK1) and polo-like kinase 1 (PLK1), which inactivates the isomerase domain and enhances protein stability, respectively^23,24^. In prostate cancer cell lines, there was a positive correlation between protein levels of DAPK1 and Pin1 but no correlation between PLK1 and Pin1 expression (Supplementary Fig. 1j, l). Analysis of clinical samples indicated that DAPK1 was expressed in CRPC and neuroendocrine prostate cancer, while PLK1 was weakly expressed in CRPC (Supplementary Fig. 1k, m).

The impact of the loss of Pin1 expression on AR transcriptional activity was evaluated in Pin1 knockdown experiments using two Pin1-targeting siRNAs that target separate regions of the human PIN1 transcript (Fig. 1c, d). Transcriptional activity of full-length AR was assessed in LNCaP cells using two well-characterized AR-driven reporter gene constructs. Androgen induction of PSA(6.1 kb)- and PB-luciferase activities were significantly decreased with reduced expression of Pin1 compared to activities measured in cells transfected with a non-targeting control siRNA (Fig. 1e, f). Pin1 knockdown did not inhibit the activities of non-AR-driven reporters, cytomegalovirus (CMV) or AP-1-luciferase reporters (Fig. 1g,h), thereby suggesting selectivity of Pin1 expression on AR transcriptional activity.

**Fig. 2:**
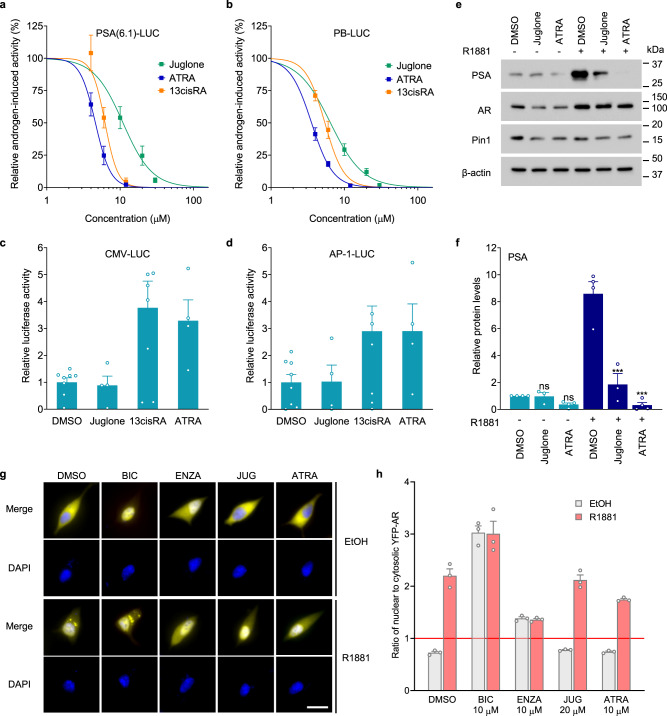
Pin1 inhibitors block the transcriptional activity of AR. **a**, **b** Inhibitory dose–response curves for juglone, ATRA, and 13cisRA on the activity of PSA(6.1 kb)- and PB-luciferase in LNCaP cells stimulated with R1881 (1 nM) for 24 h. Data shown are normalized to the induction by R1881, which was 149-fold for PSA (6.1 kb)- and 1420-fold for PB-luciferase, *n* = 4 independent experiments. (**c**, **d**) Activities of CMV and AP-1 reporters after incubating with juglone (20 µM), 13cisRA (10 µM), ATRA (10 µM), or vehicle (DMSO) for 24 h. Data shown represent the means ± s.e.m. from four independent experiments. **e** Western blot analysis of PSA, AR, and Pin1 protein levels from LNCaP cells treated with juglone (20 µM) or ATRA (10 µM), and 1 nM of R1881 or vehicle for 24 h. **f** Graph showing the quantified PSA levels after normalizing to β-actin. **g** Representative fluorescence micrographs showing the localization of YFP-AR in LNCaP cells pre-treated with the indicated compounds and stimulated with R1881 (1 nM) or vehicle (EtOH) for 2 h. The scale bar represents 20 μm. **h** YFP-AR localization was quantified by calculating the ratio of average YFP intensity in the nucleus compared to the cytosol. Scores greater than 1 indicate nuclear localization. At least 50 cells were scored for each treatment. Data shown are the normalized means ± s.e.m. from three independent experiments. Statistical significance was determined by one-way ANOVA using Holm-Sidak’s multiple comparisons test. ***P < 0.001; ns not significant.

To determine if pharmacological inhibitors of Pin1 would yield similar results to genetic knockdown of Pin1, two known inhibitors of the PPIase domain of Pin1 were employed, juglone and all-*trans* retinoic acid (ATRA). Consistent with previous reports^25,26^, both juglone and ATRA inhibited the isomerase activity of Pin1 in a dose-dependent manner (Supplementary Fig. 2a, b). 13-*cis*-retinoic acid (13cisRA) did not inhibit the isomerase activity of Pin1 (Supplementary Fig. 2c), which supports specificity of the all-*trans* conformer as expected. Consistent with the knockdown of Pin1, both pharmacological inhibitors of Pin1 reduced the induction of PSA- and PB-luciferase activity by androgen in a dose-dependent manner (Fig. 2a, b). The IC_50_ values for juglone were 10.9 µM (SE = 1.11 µM, *R*^2^ = 0.85) for PSA and 6.45 µM (SE = 1.15 µM, *R*^2^ = 0.97) for PB, whereas ATRA was more potent and had an IC_50_ of 4.60 µM (SE = 1.05 µM, *R*^2^ = 0.74) for PSA and 3.57 µM (SE = 1.05 µM, *R*^2^ = 0.91) for PB. The 13cisRA conformer was less potent than ATRA (Fig. 2a, b), with IC_50_ values of 6.22 µM and 5.42 µM for PSA and PB, respectively, which is consistent with the conversion of 13cisRA to ATRA by cellular retinoid isomerases^27^. Also consistent with Pin1 knockdown, Pin1 inhibitors displayed selectivity for blocking AR transcriptional activity without impeding the activities of the non-AR-driven CMV- and AP-1-luciferase reporters (Fig. 2c, d).

To confirm the selective effects of Pin1 inhibitors on endogenous gene expression, protein levels of PSA and transcript levels of other well-characterized target genes of full-length AR were assessed. Pin1 inhibitors blocked the androgen-induced eightfold increase in PSA protein without impacting the levels of AR protein (Fig. 2e, f). Thus, the mechanism of impeding the androgen-induced transcriptional activity of AR by Pin1 inhibitors does not involve decreased levels of AR protein. Androgen-induced KLK3/PSA, TMPRSS2, and NKX3.1 transcripts were significantly inhibited by Pin1 inhibitor (Supplementary Fig. 3a–d). The androgen-induced levels of FKBP5 transcript that were efficiently blocked by antiandrogen enzalutamide were poorly blocked by Pin1 inhibitor, thereby suggesting gene-specific responses (Supplementary Fig. 3d). In the presence of androgen, levels of AR and PIN1 transcripts were not significantly affected by either antiandrogen or Pin1 inhibitor (Supplementary Fig. 3e, f).

There is one putative Pin1-binding motif (Ser650-Pro651) within the AR hinge region, which harbors a bipartite nuclear localization signal required for AR nuclear translocation^28^ and could potentially be impacted by inhibiting Pin1. To investigate this, cells that express a YFP-AR fusion protein were treated with Pin1 inhibitors, and cellular localization of AR was determined. As expected in the absence of androgen, YFP-AR was mainly cytosolic and then predominantly nuclear following androgen treatment (Fig. 2g). In the absence of androgen, AR remained cytosolic in the presence of Pin1 inhibitors, contrary to bicalutamide and enzalutamide which increased AR nuclear localization as previously reported^9,29^ (Fig. 2g, h). Pin1 inhibitors did not prevent androgen-induced nuclear translocation of AR (Fig. 2g, h). These results suggest that the mechanism of impeding AR transcriptional activity by Pin1 inhibitors does not involve blocking AR nuclear translocation or affect the androgen-induced function of the bipartite nuclear localization signal adjacent to a potential Pin1-binding site in the hinge region.

### Targeting Pin1 inhibits transactivation of AR NTD

**Fig. 3:**
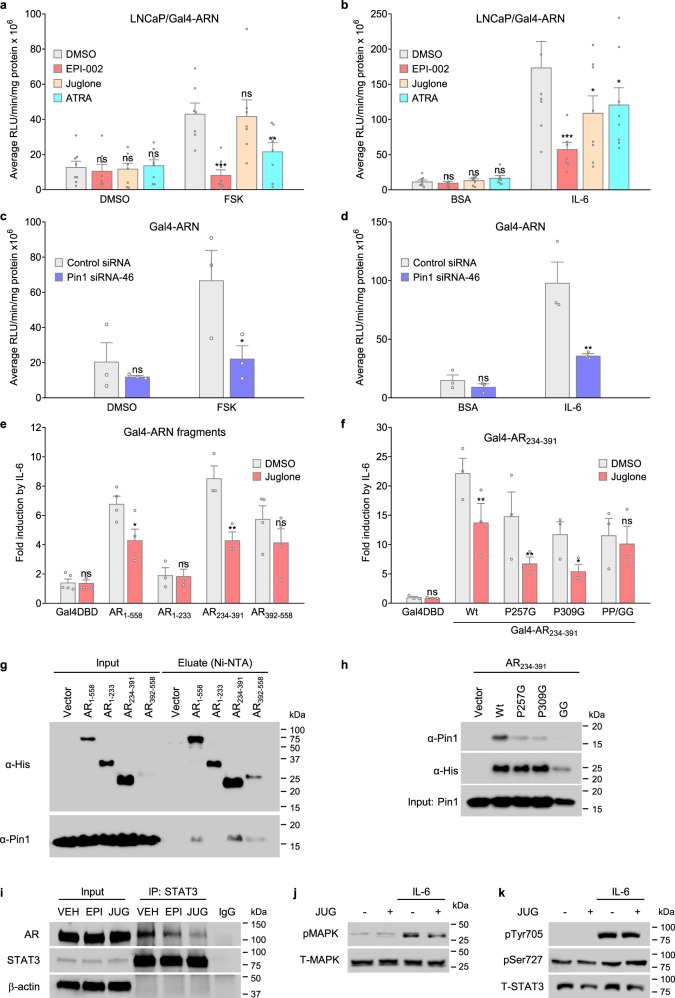
Pin1 interacts with the AR NTD and regulates transactivation. **a**, **b** Transactivation assays performed in LNCaP cells expressing a vector encoding the human AR NTD fused to a Gal4 DNA-binding domain (Gal4-ARN). Cells were treated with vehicle (DMSO), EPI-002 (25 μM), juglone (20 μM), or ATRA (10 μM), and then incubated with IL-6 (**a** 50 ng/mL) or forskolin (**b** 25 μM) for 24 h, *n* = 8 independent experiments. **c**, **d** Gal4-ARN activity in cells incubated with Pin1 siRNA or a non-targeting control siRNA and then stimulated with IL-6 or forskolin for 24 h, *n* = 3. Transactivation assays performed using (**e**) Gal4-ARN fragments (*n* = 6), or (**f**) Gal4-AR_234–391_ constructs (*n* = 3) carrying proline to glycine mutations, and treated with juglone (20 µM) and IL-6 (50 ng/mL) for 24 h. Pull-down assays performed on LNCaP cells transfected with an expression vector encoding a polyhistidine-tagged (**g**) AR NTD or (**h**) AR_234–391_ construct and then incubated with IL-6 (50 ng/mL) for 6 h. Input samples show Pin1 expression from lysates before the pull-down. **i** Co-immunoprecipitation assay showing AR protein co-immunoprecipitated with STAT3 from LNCaP cells incubated with EPI-002 (EPI, 35 μM), juglone (JUG, 30 μM), or vehicle (VEH, DMSO), and IL-6 (50 ng/mL) for 6 h. **j**, **k** Levels of phosphorylated MAPK (isoforms p44 and p42) and phosphorylated STAT3 (Tyr705 and Ser727) from LNCaP cells pre-treated with juglone (20 µM) and then stimulated with IL-6 (50 ng/mL) for 15 min. Error bars show the mean ± s.e.m. *P < 0.05, **P < 0.01, ***P < 0.001; ns not significant.

The remaining putative Pin1-binding sites are within the intrinsically disordered AR NTD that contains transcription activation units, Tau-1 and Tau-5, to mediate androgen-dependent and androgen-independent transactivation, respectively^5,30^. The loss of AR-LBD shifts the location of transcriptional activity from Tau-1 with androgen, to Tau-5 in the absence of LBD^30^. To determine whether Pin1 impacts transactivation of AR NTD, we employed a transactivation assay that consists of a construct encoding the human AR_1–558_ of the NTD fused to a Gal4DBD (Gal4-ARN) that lacks AR-LBD. Forskolin- and interleukin-6 (IL-6)-induced transactivation of AR NTD^31,32^ were both blocked by treatment with an AR NTD inhibitor, ralaniten (EPI-002, positive control), as previously reported^9,33,34^ (Fig. 3a, b). ATRA and knockdown of Pin1 expression blocked both forskolin- and IL-6-induced transactivation of Gal4-ARN, whereas juglone only inhibited IL-6-induced transactivation (Fig. 3a–d).

To map the region in the AR NTD necessary for Pin1 inhibitors to mediate an effect on AR transactivation, AR NTD fragments were tested. IL-6 induced transactivation of Gal4-AR_234–391_ and Gal4-AR_392–558_ fragments while the Gal4-AR_1–233_ fragment was not inducible and had minimal activity (Fig. 3e). Pin1 inhibitor juglone inhibited transactivation of AR 234–391 and intact AR NTD (1–558), but only had a modest effect on AR 392–558. Importantly, juglone did not inhibit the activity of Gal4DBD (empty vector) or AR 1–233. These findings suggest that inhibiting the Pin1 PPIase domain selectively blocks IL-6-induced transactivation of AR NTD by involving the region of AR encoded by amino acids 234–391.

To determine whether putative Pin1 motifs located in the AR 234–391 region (Ser256-Pro257 and Ser308-Pro309) were involved in transactivation, we assessed the activity of Gal4-AR_234–391_ constructs carrying proline to glycine mutations. IL-6 could still induce transactivation of the mutant AR 234–391 constructs (P257G, P309G, and P257G/P309G), but their fold-inductions were overall reduced by 33–48% (Fig. 3f). Juglone inhibited the P257G and P309G mutants to levels that were comparable to wild-type but had no effect on the P257G/P309G double mutant. Collectively these results suggest that both the Ser256-Pro257 and Ser308-Pro309 motifs were sufficient to regulate transactivation of AR NTD.

We performed pull-down assays to determine whether Pin1 interacted with AR NTD. Pin1 interaction was detected with AR 1–558, AR 234–391, and AR 392–558, but not with AR 1–233 (Fig. 3g). With the AR 234–391 fragment, Pin1 interaction was detected despite mutation of either putative Pin1-binding motifs (P257G or P309G), but interaction was minimal with the P257G/P309G double mutant (Fig. 3h). These results imply that Pin1 can interact with either Ser256-Pro257 or Ser308-Pro309 on AR NTD, which is consistent with transactivation assays (Fig. 3f). Co-immunoprecipitation assays confirmed interaction between endogenous full-length AR and Pin1, regardless of androgen (Supplementary Fig. 4a). We also detected interaction between Pin1 and AR-V7, following ectopic expression of AR-V7 in LNCaP cells (Supplementary Fig. 4b). Androgen-independent transactivation of AR by IL-6 signaling involves interaction between signal transducer and activator of transcription 3 (STAT3) and residues 234–558 of the AR NTD^31^. This overlaps with the region required for Pin1 interaction (Fig. 3g). Thus, we assessed if the interaction between AR and STAT3 would be impacted by a Pin1 inhibitor. Endogenous STAT3 complexes were co-immunoprecipitated from LNCaP cells treated with juglone and IL-6. Ralaniten was included as a positive control for inhibiting STAT3 interaction with AR NTD^33^. Juglone reduced the amount of AR interacting with STAT3 by ~50%, which was similar to the amount decreased by ralaniten (Fig. 3i**)**. Juglone did not prevent phosphorylation of MAPK (isoforms p44 and p42) or STAT3 (Tyr705 and Ser727) (Fig. 3j, k). These data suggest that targeting Pin1 inhibits the interaction between the AR NTD and STAT3 by a mechanism that does not involve preventing IL-6-induced phosphorylation of STAT3 or MAPK.

### Pin1 inhibitors block the proliferation of prostate cancer cells driven by full-length AR and AR-Vs

**Fig. 4:**
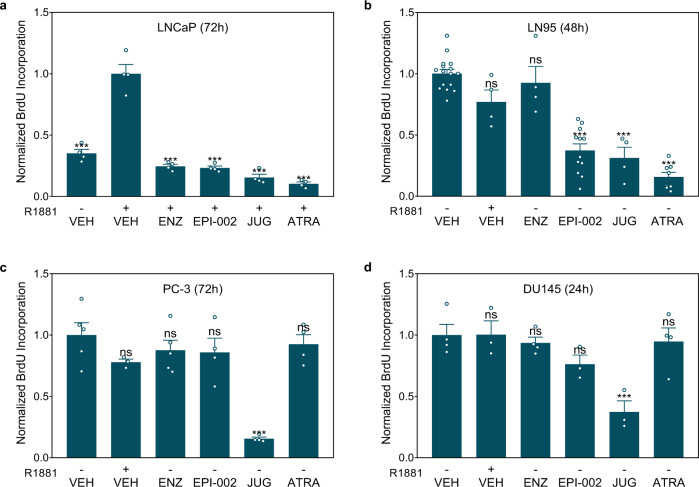
ATRA suppresses prostate cancer cell proliferation driven by full-length AR and AR-Vs. **a**–**d** Proliferation assays showing the effect of AR inhibitors (ENZ, 10 µM; and EPI-002, 25 µM) and Pin1 inhibitors (JUG, 20 µM; and ATRA, 10 µM) on BrdU incorporation of the indicated prostate cancer cell lines: LNCaP (**a** androgen-sensitive); LN95 (**b** androgen-independent); PC-3 and DU145 (**c** and **d**, AR non-reliant). Cells were treated with the inhibitors and stimulated with 1 nM of R1881 (+) or vehicle (−) for 24 to 72 h. Each data point represents the average from an independent experiment where the error bars show the mean ± s.e.m. Statistical significance was determined by one-way ANOVA using Dunnett’s multiple comparisons test. *P < 0.05, **P < 0.01, ***P < 0.001; ns not significant.

AR drives the proliferation of most prostate cancer cells. To determine whether blocking AR transcriptional activity by Pin1 inhibitors leads to decreased proliferation, BrdU incorporation assays were utilized in a battery of cell lines. Androgen-induced proliferation of LNCaP cells depends on functional full-length AR. LN95 cells express full-length AR and AR-Vs, including AR-V7, but their proliferation is driven by AR-Vs and is not increased by androgen and thus are resistant to AR-LBD inhibitors. PC-3 and DU145 cells are devoid of functional AR and are androgen-independent. The effects of Pin1 inhibitors were compared to enzalutamide (AR-LBD inhibitor) and ralaniten (AR-NTD inhibitor) as controls. Pin1 inhibitors attenuated androgen-induced proliferation of LNCaP cells similar to validated AR antagonists (Fig. 4a). Pin1 inhibitors also attenuated the proliferation of LN95 cells along with ralaniten, whereas the AR-LBD inhibitor enzalutamide had no effect (Fig. 4b). These results are consistent with the proliferation of this cell line mediated by truncated AR-Vs that lack AR-LBD and with Pin1 regulating AR NTD. Of the Pin1 inhibitors tested, ATRA had better selectivity for blocking AR-dependent proliferation compared to juglone and did not impact the proliferation of either PC-3 or DU145 cells (Fig. 4c, d). Collectively these results support that ATRA has selectivity for blocking proliferation mediated by full-length AR or AR-Vs. Based on these findings, we focused on ATRA as a pharmaceutical inhibitor of Pin1 since its effects were consistent with knockdown of Pin1, whereas juglone has known cytotoxic effects on non-specific targets involved in mitosis^35^.

### Pin1 inhibitors and knockdown block AR-V7 transcriptional activity

**Fig. 5:**
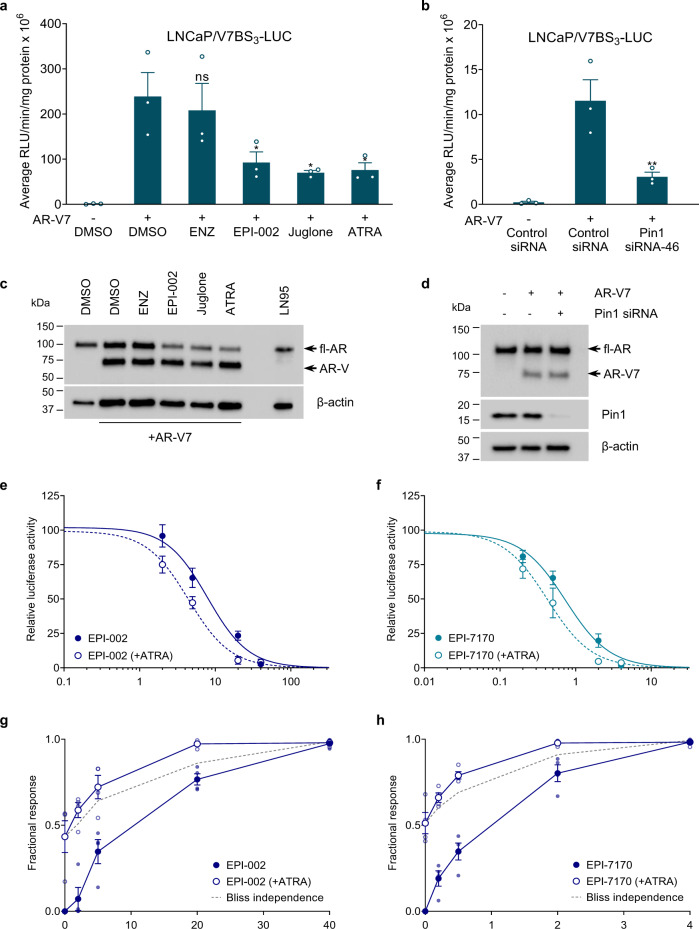
ATRA enhances the inhibitory response of EPI compounds. **a**, **b** AR-V7 transcriptional activity in LNCaP cells ectopically expressing AR-V7 and transfected with a reporter regulated by AR-V7-binding sites (V7BS_3_-luciferase). Cells were incubated with **a** enzalutamide (10 µM), EPI-002 (25 µM), juglone (20 µM), ATRA (10 µM), or vehicle (DMSO) for 24 h, or either **b** Pin1 siRNA or a non-targeting control siRNA for 48 h (*n* = 3 independent experiments). **c**, **d** Western blots showing the levels of ectopic AR-V7 and the efficiency of the Pin1 knockdown. The inhibitory dose–response of **e** EPI-002 and **f** EPI-7170 for inhibiting the androgen-induced PSA(6.1 kb)-luciferase activity in LNCaP cells, in the presence and absence of 5 µM of ATRA. Data shown are normalized to the fold-induction by R1881, which was approximately 82-fold at 24 h. ATRA shifted the inhibitory dose–response curves of EPI-002 and EPI-7170 to the left, thereby lowing their IC_50_ values. Graphs show the fractional responses of **g** EPI-002 and **h** EPI-7170 as monotherapies and combination therapy with 5 µM of ATRA, compared to the predicted additive effect determined by the Bliss independence model (*n* = 4). Data shown represent the means ± s.e.m. *P < 0.05, **P < 0.01; ns not significant.

Pin1 interacted with the AR NTD (Fig. 3g) and reduced expression or activity of Pin1 impeded both AR NTD transactivation (Fig. 3a–d) and AR-V-driven proliferation of LN95 cells (Fig. 4b). To directly determine if Pin1 is required for AR-V7 transcriptional activity, the V7BS_3_-luciferase reporter that is driven by three tandem repeats of an AR V7-specific promoter element from the *UBE2C* gene^36^ was tested. As expected, V7BS_3_ luciferase was highly induced by ectopic expression of AR-V7, which could not be blocked by AR-LBD inhibitor enzalutamide (Fig. 5a). Consistent with blocking AR NTD transcriptional activity, ralaniten, Pin1 inhibitors, and Pin1 knockdown all decreased AR-V7-induced reporter activity (Fig. 5a, b). Western blot analyses revealed similar levels of AR-V7 between the different treatments and siRNAs (Fig. 5c, d). Collectively, these results imply that the isomerase activity of Pin1 impacts AR-V7 transcriptional activity through the AR NTD.

### Combination therapy with ralaniten and targeting Pin1

Developing better treatments for CRPC is urgently needed, especially one that inhibits the AR NTD to block the transcriptional activities of AR-Vs. A double strike against the AR NTD by combination therapy targeting Pin1 together with ralaniten was therefore investigated as a possible therapeutic approach. Since ATRA is approved clinically for other indications with known toxicity profiles, we focused on this compound in combination with ralaniten and a second-generation analog EPI-7170 that has improved potency^37^. The impact of a suboptimal concentration of ATRA (5 μM) on dose–response curves for ralaniten and EPI-7170 for inhibiting androgen-induced PSA(6.1 kb)-luciferase activity was determined. The IC_50_ of ralaniten for PSA(6.1 kb) luciferase was 7.91 ± 1.15 μM (*R*^2^ = 0.94), and the curve shifted to the left with ATRA and an improved IC_50_ of 4.45 ± 1.28 µM (*R*^2^ = 0.83) (Fig. 5e). A similar trend was observed with EPI-7170, which had an IC_50_ of 0.74 ± 1.12 μM (*R*^2^ = 0.96) as monotherapy and an IC_50_ of 0.42 ± 1.16 μM (*R*^*2*^ = 0.93) with ATRA (Fig. 5f). For both ralaniten compounds, the inhibitory responses in combination with ATRA exceeded the predicted additive effects determined by the Bliss independence model^38^ (Fig. 5g, h). Excluding the highest concentrations (40 µM of ralaniten and 4 µM of EPI-7170), which inhibited ~98% of reporter activity, the mean combination index (CI) for ralaniten and EPI-7170 was 0.86 and 0.91, respectively. This signifies that the ralaniten compounds were moderately synergistic with ATRA.

### Effect of Pin1 inhibitors and combinations on the cell cycle

**Fig. 6:**
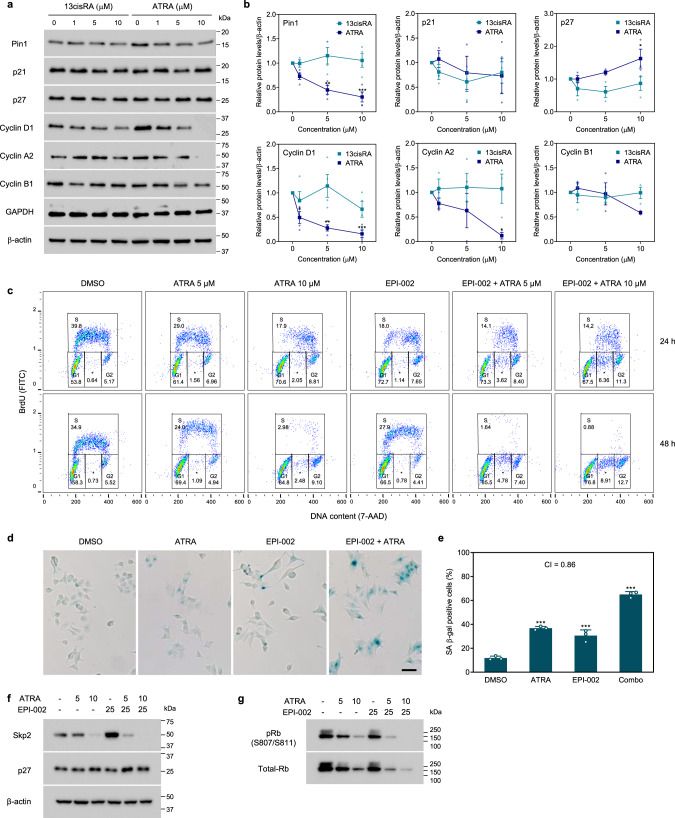
ATRA and EPI are synergistic in blocking cell cycle progression. **a** Expression of cell cycle proteins from lysates of LN95 cells treated with 0–10 µM of ATRA or 13cisRA. **b** Graphs show the quantified levels of the indicated proteins after normalizing to β-actin (*n* = 4). **c** Cell cycle analysis of LN95 cells treated with vehicle (DMSO), ATRA (5 or 10 μM), EPI-002 (25 μM), or combinations for 24 or 48 h in media supplemented with 1.5% CSS. BrdU-negative S phase cells are denoted by asterisk (*). **d** Staining of SA β-gal activity performed on LN95 cells after incubating with ATRA (10 μM), EPI-002 (25 μM), ATRA (10 μM), or a combination for 3 days. Original magnification: ×100. The scale bar represents 50 µm. **e** The graph shows the percentage of β-gal-positive cells scored from at least 1000 cells for each treatment group from three independent experiments. **f**, **g** Representative immunoblots showing the expression of indicated cell cycle proteins after incubating with combination therapy. Data shown are the means ± s.e.m. from 3–4 independent experiments. Statistical significance was determined by ANOVA using the Dunnett’s multiple comparisons test. *P < 0.05, **P < 0.01, ***P < 0.001.

Many well-defined Pin1 substrates are mitotic proteins that are involved in regulating cell cycle progression^39^. AR-V7 is reported to regulate the expression of a distinct subset of cell cycle-related genes^40^, including *UBE2C*. To determine whether ATRA alters the expression of cell cycle proteins, we performed western blot analyses from LN95 cells treated with ATRA (0–10 µM) or the inactive 13cisRA conformer. ATRA binds directly to the catalytic pocket of Pin1, which inhibits the PPIase domain and induces degradation of Pin1 protein^25^. Consistent with this mechanism, a dose-dependent decrease in Pin1 levels was measured with ATRA (Fig. 6a,b). 13cisRA did not impact the cellular levels of Pin1, thereby supporting the specificity of ATRA for Pin1. ATRA significantly decreased the expression of cyclin D1 and cyclin A2 (G_1_ and S cyclins, respectively), but not cyclin B1 (M cyclin), and selectively increased the levels of cyclin-dependent kinase (CDK) inhibitor p27^Kip1^ but not p21^Cip1^ (Fig. 6b). Together these findings demonstrate the on-target inhibition of Pin1 by ATRA and reveal a perturbation of proteins involved in facilitating the G_1_/S transition.

Combinations of ATRA and EPI were tested for their effect on the cell cycle by flow cytometry. Analysis of BrdU incorporation and DNA content showed that 35–40% of LN95 cells were active in S phase with vehicle (Fig. 6c, column 1). ATRA reduced the S phase population in a dose- and time-dependent manner, which was accompanied by a concomitant increase in G_1_ cells (Fig. 6c, columns 1–3). Combinations of ATRA (5 and 10 μM) with ralaniten further reduced the S phase, where ralaniten with 5 μM of ATRA showed a comparable response on S phase to the combination with 10 μM of ATRA (Fig. 6c, columns 4–6). These analyses suggest that ATRA and ralaniten were synergistic in blocking the cell cycle, possibly by arresting cells in G_1_ and blocking the transition to S phase. Consistent with this notion, ATRA inhibited the ability of LN95 cells to form colonies in a dose-dependent manner (Supplementary Fig. 5a). Ralaniten also significantly reduced the number of colonies, and combination with 10 µM of ATRA had an improved response compared to either monotherapy (Supplementary Fig. 5b). Together these results suggest that combination therapy of ATRA and ralaniten blocks the cell cycle and may irreversibly impair cell division.

Since combination of ATRA and ralaniten markedly increased the number of cells in G_1_, we sought to determine whether the response was associated with senescence by measuring senescence-associated β-galactosidase (SA β-gal) activity^41,42^. ATRA significantly increased the percentage of SA β-gal-positive cells for LNCaP and LN95 cells, but not for PC-3 and DU145 cells (Supplementary Fig. 6a, b). Expression of p16^INK4A^, a marker of replicative senescence and aging, was not induced by ATRA and was overall weakly expressed (Supplementary Fig. 6c). LN95 cells treated with a combination of ralaniten and ATRA showed intense SA β-gal staining and developed a morphology that is associated with senescent cells (appearing larger and more flattened) (Fig. 6d). Approximately 65% of cells treated with combination therapy stained positive for SA β-gal, which was higher than for ralaniten (31%) or ATRA (37%) monotherapies (Fig. 6e). The CI of ATRA and ralaniten for producing a senescence response was 0.86, which indicates that the combination is slightly synergistic. Western blot analyses revealed a loss of F-box protein Skp2 (S-phase kinase-associated protein 2) and upregulation of CDK inhibitor p27^Kip1^ by combination of ralaniten and ATRA (Fig. 6f). These results are consistent with the induction of a senescence response in prostate cells that depend on AR function for proliferation^43,44^. The levels of retinoblastoma protein (Rb) and phospho-Rb (serine residues 807 and 811) were also decreased by both monotherapies and combination therapy (Fig. 6g). Collectively, these findings suggest that targeting the AR NTD with ATRA and ralaniten may induce G_1_ arrest and senescence in prostate cancer cells that express AR-Vs, by a mechanism involving the Skp2/p27^Kip1^ pathway and Rb inactivation but not requiring p16^INK4A^.

### Combination therapy inhibits the in vivo growth of enzalutamide-resistant CRPC xenografts

**Fig. 7:**
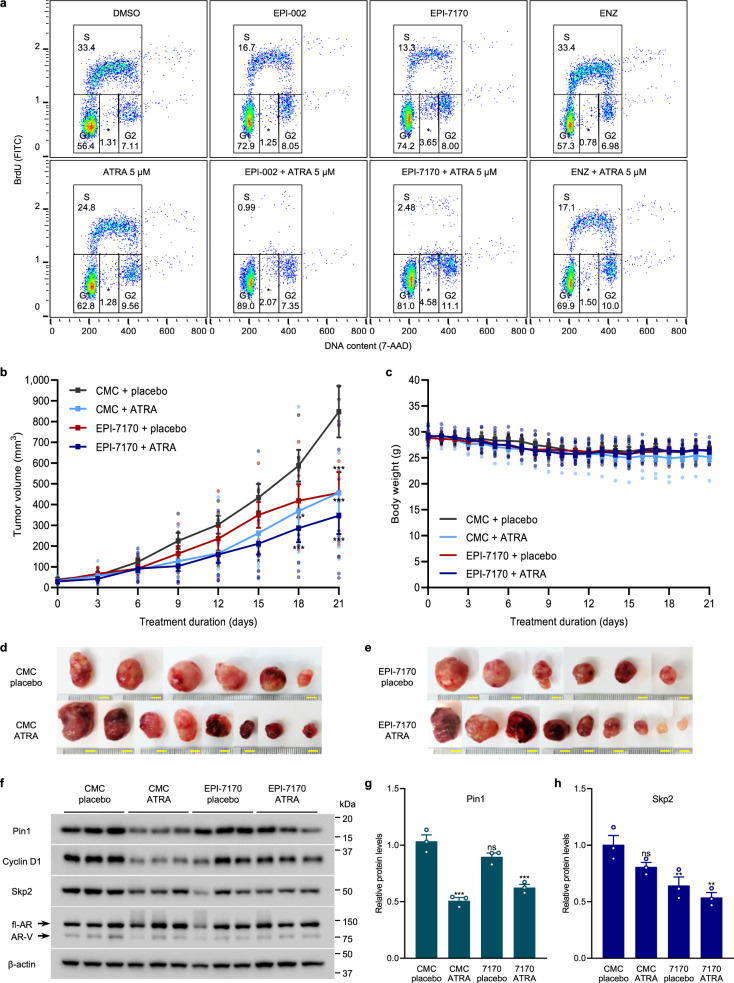
Combination therapy with ATRA and EPI reduces the in vivo growth of CRPC xenografts driven by AR-Vs. **a** Cell cycle analysis by flow cytometry of LN95-D3 subline incubated with monotherapy and combination therapy of ATRA (5 or 10 μM) with EPI-002 (25 μM), EPI-7170 (5 μM), or enzalutamide (ENZ, 10 μM), for 48 h in media supplemented with 1.5% CSS. **b** Growth curves of LN95-D3 tumors established in castrated male NSG mice bearing 21-day release placebo or ATRA (5 mg) pellets and treated daily with either vehicle (CMC) or EPI-7170 (30 mg/kg/d), where pellets were implanted on day 0 and oral dosing began on day 3. **c** Graph showing the average body weight of animals for each treatment group during the study. **d**, **e** Images of the xenograft tumors harvested at the end of the study. Scale bars represent 5 mm (**f**) western blot analysis showing the expression of Pin1, Cyclin D1, Skp2, and AR from representative tumors. Quantification of the data is shown for **g** Pin1 and **h** Skp2, where the error bars represent the mean ± s.e.m. Statistical significance was determined by one- and two-way ANOVA using Dunnett’s multiple comparisons test. *P < 0.05, **P < 0.01, ***P < 0.001; ns not significant.

To evaluate the efficacy of combination therapy with ATRA and ralaniten in vivo, we employed LN95-D3 cells (clonal subline)^45^. Consistent with the parental LN95 cells, enzalutamide had no effect on the cell cycle distribution of the LN95-D3 subline in the absence of androgen, whereas treatment with ATRA or ralaniten compounds reduced the S phase and increased the G_1_ population (Fig. 7a, columns 1–3). Furthermore, combination therapy of ATRA with either ralaniten or EPI-7170 further decreased the S phase. This synergy was specific to combinations of ATRA and ralaniten compounds and was not apparent with combination of ATRA and enzalutamide (Fig. 7a, column 4). These data support that proliferation of LN95-D3 cells was resistant to enzalutamide but remains sensitive to ralaniten compounds that target the AR NTD, which was consistent with the cells being driven by AR-Vs.

A pilot study was conducted to study the effects of ATRA monotherapy on the growth of LN95-D3 CRPC xenografts. The growth of LN95-D3 tumors was markedly reduced in mice bearing ATRA slow-release pellets compared to placebo (Supplementary Fig. 7a). On-target activity of ATRA was validated by a decrease in Pin1 levels in xenografts from mice treated with ATRA pellets compared to placebo (Supplementary Fig. 7b**)**. Moreover, we measured levels of serum PSA, a widely used and well-characterized clinical biomarker for CRPC progression and patient responses to therapy. PSA expression in sera was reduced on average by 27% in animals treated with ATRA compared to placebo, albeit the responses varied between animals and did not reach statistical significance (Supplementary Fig. 7c). Notably, there was a positive correlation between serum PSA and tumor volume (Supplementary Fig. 7d), which implies that serum PSA was a reliable surrogate marker for tumor progression of the LN95-D3 xenograft model.

Based on these findings, a larger study was undertaken to compare monotherapies to combination therapy of ATRA and EPI-7170 in castrated mice bearing LN95-D3 xenografts. The animals were implanted with a 21-day release placebo or ATRA (5 mg) pellet and then treated daily with either EPI-7170 (30 mg/kg) or vehicle (CMC). After 21 days of treatment, tumor growth was significantly reduced in the animals treated with ATRA, EPI-7170, and combination, relative to the vehicle/placebo-control (Fig. 7b). The combination therapy was superior to individual monotherapies in reducing the final tumor volume and reached statistical significance earlier than the monotherapies. There were no significant differences in body weight of the animals during the study, and the treatments were well-tolerated (Fig. 7c).

**Fig. 8:**
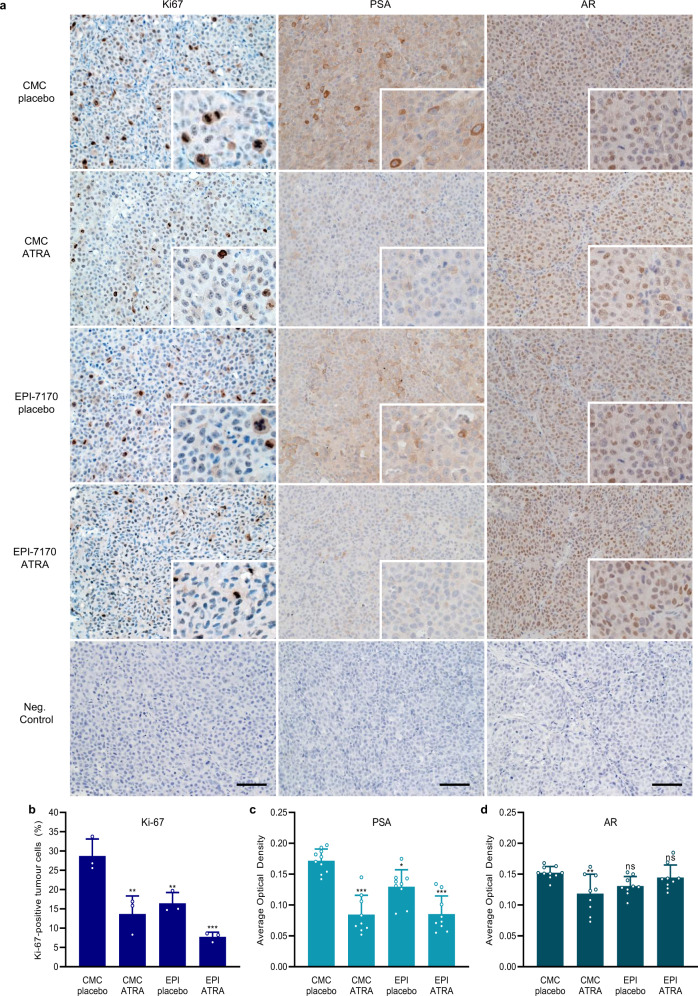
Immunohistochemistry of LN95-D3 xenografts. **a** Representative images showing the histomorphology and expression of Ki-67, PSA, and AR from harvested tumors. Scale bars represent 100 µm. Statistical analysis for **b** the percentage of Ki-67-positive tumor cells and for the expression of **c** PSA and **d** AR. Error bars represent the mean ± s.d. Statistical significance was determined by one-way ANOVA using the Dunnett’s multiple comparisons test. *P < 0.05, **P < 0.01, ***P < 0.001; ns not significant.

Xenograft tumors harvested at the end of the study were variable in appearance, but several of the tumors from the combination group were remarkably reduced in size and white in appearance (Fig. 7d, e). Western blot analyses of tumor lysates confirmed the on-target activity of ATRA with decreased levels of Pin1 and its target cyclin D1 (Fig. 7f, g). Consistent with in vitro data (Fig. 6f), tumor levels of Skp2 were reduced by EPI-7170, combination therapy, and to a modest extent by ATRA (Fig. 7f, h). The expression of full-length AR and AR-Vs varied between tumors, but overall levels and the ratio of AR-V to full-length AR were not significantly impacted by treatment. Analysis of whole blood collected throughout the study revealed a 50% decrease in serum PSA from animals treated with combination therapy compared to the control (Supplementary Fig. 8), where 6 out of 9 animals on combination therapy showed stable or regression of PSA during the study (Supplementary Fig. 8d). Sections of harvested xenograft were assessed by immunohistochemistry for proliferation antigen Ki-67 and expression of PSA and AR. Consistent with ATRA and EPI-7170 decreasing tumor volume, ATRA, EPI-7170, and combination therapy decreased the number of Ki-67-positive tumor cells in xenografts (Fig. 8a, b). PSA expression was also decreased by ATRA, EPI-7170, and combination therapy, compared to the control (Fig. 8a, c). The levels of AR protein were similar in xenografts from animals treated with EPI-7170 or combination therapy and was decreased slightly by ATRA (Fig. 8a, d). Collectively, these in vivo data with in vitro data support that combination therapy of a ralaniten analog (EPI-7170) with a Pin1 inhibitor (ATRA) inhibits the growth of CRPC tumors driven by AR-Vs.

## Discussion

Increasing evidence suggests that AR-Vs underlie resistance of CRPC to second-generation AR-LBD inhibitors^2,46–48^. This has led to an interest in finding therapies that target the AR NTD. Unfortunately, this domain is intrinsically disordered. Only one drug, ralaniten, has been tested clinically that directly binds to any intrinsically disordered target, thereby emphasizing the hurdles in developing drugs to these difficult targets. Ralaniten showed clinical signs of efficacy but had poor pharmacokinetics that led to excessive pill burden^49^. A second-generation ralaniten analog with improved stability, EPI-7386, is currently in Phase I evaluation. Ralaniten binds residues 354– 448^7^ predominantly within Tau-5 (residues 360–485). In response to androgen, transcriptional activity of full-length AR resides in Tau-1 (residues 101–370). The loss of AR-LBD with AR-Vs shifts the site of transcriptional activity from Tau-1 with androgen to Tau-5 in the absence of androgen^30^. Multiple hits against the AR NTD that encompass both Tau-1 and Tau-5 are proposed to yield better therapeutic responses for CRPC that express both full-length AR and AR-Vs^6^.

Pin1 motifs are predicted across the entire AR NTD and could potentially impact transactivation of AR NTD. This *cis/trans*-proline isomerase is frequently elevated in prostate cancer and associated with disease recurrence^10–12^. We found that Pin1 interacted with AR NTD predominantly within Tau-1 (residues 234–391) and that genetic and pharmacological inhibition of Pin1 selectively disrupted several key aspects of AR signaling. These included inhibition of: (1) transcriptional activities of full-length AR and AR-V7; (2) transactivation of AR NTD; (3) protein–protein interactions; and (4) AR- and AR-V-dependent proliferation of prostate cancer cells. A combination of pharmacological Pin1 inhibitor with ralaniten compounds was synergistic for blocking cell cycle progression and promoted senescence of enzalutamide-resistant LN95 cells whose proliferation is driven by AR-Vs. Consistent with in vitro data, this combination therapy had superior antitumor effects in vivo compared to monotherapies.

Here we provide evidence that genetic and pharmacological inhibition of Pin1 selectively impeded AR transcriptional activity and AR-driven proliferation. Support for selectivity was drawn from clinical data supporting the association between PIN1 levels and the levels of AR and its target genes KLK3/PSA, TMPRSS2, and NKX3.1. Non-AR-driven reporters were not blocked by Pin1 inhibitors or knockdown contrary to reporters driven by full-length AR and AR-V7. Gene expression analyses showed a sensitivity of AR-regulated genes to inhibiting Pin1 activity. On-target activity against Pin1 was measured in harvested CRPC xenografts. Similarly, inhibiting Pin1 induced known biological responses that occur with the loss of AR function that included attenuating progression from G_1_ to S phase of the cell cycle and increased cellular senescence^50^.

Functional AR is required for the progression of cells from G_1_ to S phase of the cell cycle^51,52^. Consistent with these reports, both ralaniten compounds and ATRA decreased the S phase population and increased G_1_, whereas enzalutamide had no effect on AR-V-driven proliferation. G_1_ arrest was accompanied by increased levels of p27^Kip1^ and the loss of cyclins D1 and A2 by ATRA which were expected in response to blocking AR function^52^. Similarly, ATRA and ralaniten both increased cellular senescence with concomitant losses of Skp2 and pRb which are known responses to a loss of AR function^50,51^. A combination of ATRA with the more potent ralaniten analog EPI-7170 was more effective than the individual monotherapies in inhibiting the in vivo growth of LN95-D3 xenograft tumors, reducing serum PSA, and was not toxic to the animals. This xenograft model expresses PSA in castrated animals and demonstrated robust growth in vivo with a tumor volume doubling time of approximately 3 days; therefore, it is a representative model for aggressive, AR-V7-positive CRPC. ATRA (tretinoin) is the only clinically used drug known to inhibit Pin1 and is FDA-approved to treat acute promyelocytic leukemia. These studies overall support that repurposing ATRA as a combination therapy with a ralaniten analog could provide a significant near-term clinical impact for the treatment of CRPC.

## Methods

### Cell culture

Cell lines were obtained from the following sources: LNCaP cells from Dr. Leland Chung (Cedars-Sinai Medical Centre, Los Angeles, CA); LNCaP95 (LN95) cells from Dr. Stephen Plymate (University of Washington, Seattle, WA); VCaP cells and PC-3 cells from the American Type Culture Collection (Manassas, VA); and DU145 cells from Dr. Victor Ling (British Columbia Cancer Agency, Vancouver, BC). LNCaP, VCaP, PC-3, and DU145 cells were authenticated by short tandem repeat analysis and tested to ensure they were mycoplasma-free by DDC Medical (Fisher Scientific, Ottawa, ON). Cell lines were maintained in the following culture media: LNCaP cells in RPMI-1640 media with 5% fetal bovine serum (FBS); LN95 in RPMI-1640 media with 10% charcoal-stripped serum (CSS); PC-3 and DU145 cells, in DMEM (Invitrogen, Carlsbad, CA) with 5% and 10% FBS, respectively, and supplemented with 2 mM l-glutamine and 1 mM of sodium pyruvate; VCaP cells in DMEM (Sigma-Aldrich, St. Louis, MO) with 10% FBS, 2 mM l-glutamine, and 1 mM sodium pyruvate. All cell lines were maintained at 37 °C in a humidified incubator containing 5% CO_2_ for not more than ten passages. Cell lines were routinely tested with the Venor GeM Mycoplasma Detection Kit (Sigma-Aldrich) to ensure that they were mycoplasma-free.

### Compounds and reagents

ATRA, 13-*cis*-retinoic acid (13cisRA), and juglone were purchased from Sigma-Aldrich. Ralaniten (EPI-002) was provided by NAEJA (Edmonton, AB) and second-generation analog EPI-7170 was synthesized by Dr. Raymond Andersen (University of British Columbia). Enzalutamide was purchased from OmegaChem (Lévis, Québec) and bicalutamide was a gift from Dr. Marc Zarenda (AstraZeneca, Cambridge, England). Synthetic androgen methyltrienolone (R1881) was purchased from Perkin-Elmer (Waltham, MA), IL-6 from R&D Systems (Minneapolis, MN), and forskolin from EMD Millipore (Burlington, MA). Antibodies against various proteins were from the following sources, mouse monoclonal antibodies: Pin1 (8C10), Cyclin D1 (DCS-6) from Santa Cruz Biotechnology; STAT3 (124H6), Cyclin A2 (BF683), Rb (4H1) from Cell Signaling Technology; GAPDH (6C5) from Invitrogen; β-actin (A5441), polyhistidine (HIS-1) from Sigma-Aldrich; rabbit monoclonal: DAPK1 (#3008), PLK1 (208G4), Cyclin B1 (D5C10), p16 INK4a (D3W8G), p21 Waf1/Cip1 (12D1), p27 Kip1 (D69C12), PSA/KLK3 (D11E1), phospho-Rb (Ser807/811; D20B12) from Cell Signaling Technology; rabbit polyclonal: AR (N-20) from Santa Cruz Biotechnology; phospho-STAT3 (Tyr705; D3A7), p44/42 MAPK (9102), phospho-p44/42 MAPK (Thr202/Tyr204), Skp2 (D3G5) from Cell Signaling Technology.

### Transfections and luciferase assays

The PSA(6.1 kb)-luciferase and probasin-luciferase plasmids and transactivation assays for AR-(1–558)-Gal4DBD, AR-(1–233)-Gal4DBD, AR-(234–391)-Gal4DBD, AR-(392–558)-Gal4DBD have been previously described^8,31,32^. All point mutations were made by site-directed mutagenesis using QuikChange II (Agilent Technologies, Santa Clara, CA). Mutations were verified by Sanger sequencing from the UBC Nucleic Acid Protein Service Unit. Transfections with siRNA were performed using 10 nM of Pin1-targeting siRNA (s10544, s10546) or non-targeting control siRNA (4390843) with lipofectamine RNAiMAX reagent diluted in Opti-MEM media (Invitrogen). For reporter assays, transfections were performed in serum-free media with Lipofectin (Invitrogen) or FugeneHD (Promega, Madison, WI). Luciferase activity was measured for 10 s with the Luciferase Assay System (Promega) and normalized to total protein concentration determined by Bradford assay.

### Pin1 inhibition assay

The effect of inhibitors on Pin1 isomerase activity was evaluated using a fluorometric Pin1 Assay Kit (AnaSpec, Fremont, California), according to the manufacturer’s protocol. In brief, compounds (0–50 µM) were incubated with 500 ng of recombinant human Pin1 protein (ab51230; Abcam, Cambridge, UK) in a black 96-well, clear-bottom plate (BD Biosciences, San Jose, CA), and the reaction was started by adding the fluorogenic substrate. Fluorescence intensity was measured with excitation wavelength at 490 nm and emission wavelength at 520 nm on an Infinite M1000 plate reader (Tecan, Männedorf, Switzerland) at 37 °C. Measurements were recorded every 5 min for up to 1 h.

### Immunoprecipitation and western blotting

Immunoprecipitation of STAT3 complexes has been previously described^33^. In brief, cell lysates were precleared with a non-specific rabbit IgG antibody and immunoprecipated with an antibody against a STAT3 (124H6). For the interaction of full-length AR and Pin1, co-immunoprecipitation assays were performed from LNCaP cells treated with androgen (R1881, 1 nM) or vehicle for 3 h. Approximately 500 µg of whole-cell lysate was precleared with a non-specific rabbit IgG antibody, incubated with 1 µg of Pin1 (H-123) antibody for 1 h at 4 °C on a rotating rack, and then incubated for an additional hour with 50 µL of µMACS protein A/G magnetic beads (Miltenyi Biotec, Bergisch Gladbach, Germany). Pin1 was bound to µ columns (Miltenyi Biotec), washed four times with lysis buffer, and then eluted with SDS sample buffer. For western blot analyses, membranes were blocked with 5% non-fat milk in phosphate-buffered saline containing 0.1% Tween-20 (PBS-T) and then incubated overnight at 4 °C with primary antibody (1:200–5000) in PBS-T containing 5% non-fat milk or 2% BSA. After incubation with the primary antibody, the membrane was washed three times with TBS-T, incubated with a horseradish peroxidase-conjugated antibody secondary antibody (1:5000–10,000) for 1–2 h at room temperature, and washed three times with PBS-T and two more times with PBS before chemiluminescence was detected with ECL Prime Reagent (GE Healthcare Life Science, Mississauga, ON). Western blot images were captured using the ChemiDoc MP Imaging System (Bio-Rad Laboratories, Hercules, CA) and quantified using ImageJ software.

### Pull-down assays

LNCaP cells were transfected with 1 μg of Pin1 plasmid and 8–11 μg of an expression plasmid encoding his-tagged AR-(1–558), AR-(1–233), AR-(234–391), or AR-(392–558). The following day, cells were treated with compounds and stimulated with IL-6 (50 ng/mL) for 6 h. When harvesting, cell pellets were washed in PBS, flash-frozen in liquid nitrogen, and stored at −80 °C. For protein extraction, thawed cell pellets were resuspended in 1 mL of lysis buffer (50 mM HEPES, 150 mM NaCl, 10 mM imidazole, and 0.5% Triton X-100). The lysates were passed several times through a 28 gauge needle and then cleared by centrifugation. Lysates were incubated with 200 μL of Ni-NTA agarose beads for 1 h at 4 °C, washed twice with HEPES/NaCl buffer (pH 8.0) containing 60 mM imidazole, and eluted with HEPES/NaCl buffer (pH 8.0) containing 300 mM imidazole. The eluted samples were resolved on a 12.5% SDS-PAGE along with input samples (containing 10% of starting cell lysate material) and analyzed by probing for Pin1 (8C10) and polyhistidine (HIS-1).

### Fluorescence microscopy

AR translocation assays were performed by a modified protocol from Myung et al.^9^. Approximately 2.8 × 10^5^ LNCaP cells were grown on coverslips in six-well plates and transiently transfected with 2 μg of an expression vector encoding a YFP-AR fusion protein. On the following day, cells were pre-treated with the indicated compounds for 1 h and stimulated with R1881 (1 nM) or a vehicle containing ethanol for 3 h. After incubating with compounds, cells were fixed with 4% paraformaldehyde, washed twice with PBS, and then counterstained with DAPI. Slides were examined on an Axiovert 200 Fluorescence Microscope (Zeiss, Oberkochen, Germany). The ratio of the YFP signal in the nucleus relative to the cytoplasm was quantified with ImageJ software. For each image, background fluorescence was measured and subtracted from experimental values. A total of at least 50 cells from nine images containing six or more cells per field were quantified to determine the nuclear to cytoplasmic YFP ratio for each treatment group.

### BrdU incorporation assay

BrdU incorporation was determined with the colorimetric BrdU ELISA kit (Roche Diagnostics), according to the manufacturer’s protocol. In brief, cells were seeded in 96-well plates (LNCaP, 5000/well; LN95, 8000/well; PC-3 and DU145, 4000/wells) in media with reduced serum (0.5–1%). On the following day, cells were pre-treated with test compounds for 1 h and then incubated with 0.1 nM of R1881. After the indicated incubation times, cells were labeled with 10 μM of BrdU for 2 h and then fixed for the assay. BrdU incorporation was quantified with a VersaMax ELISA microplate reader (Molecular Devices, San Jose, CA) by measuring absorbance at 370 nm with a reference wavelength of 492 nm.

### Transcriptional reporter assay for AR-V7

The V7BS_3_-luciferase plasmid, which contains three tandem repeats of an AR-V7-specific promoter element of the *UBE2C* gene, was a gift from Dr. Stephen Plymate (University of Washington) and has been described^36^. LNCaP cells seeded in 24-well plates were co-transfected with the V7BS_3_-luciferase reporter (0.25 μg/well), an expression vector encoding AR-V7 (0.2 μg/well), and a filler plasmid (pGL4, 0.3 μg/well). On the following day, the cells were treated with the indicated compounds for 24 h. Luciferase activity was measured for 10 s using the Luciferase Assay System (Promega) and the data were normalized to total protein concentration determined by a Bradford assay. Western blot analyses were performed to verify the expression of AR-V7.

### Cell cycle analysis by flow cytometry

Cells were grown in 10 cm dishes in 10% CSS for 3 days before the media was changed to 1.5% CSS and ATRA and/or EPI-002 were added at the indicated concentrations. After treatments, the cells were labeled with BrdU (10 μM) for 2 h and then trypsinized, washed, and fixed in 70% EtOH/PBS at −20 °C. Approximately 2.5–5.0 × 10^5^ of fixed cells were labeled with fluorescein (FITC)-conjugated anti-BrdU antibody (B44; BD Biosciences) and the DNA content was stained with 7-AAD (Sigma). The data were collected with a BD FACSCalibur flow cytometer (BD Biosciences). FITC and 7-AAD fluorescence were detected in the FL1 and FL3 channels, respectively, using CellQuest Pro and analyzed by FlowJo V10 software (Ashland, Oregon). Pre-gating was performed on all samples by plotting FL3-W × FL3-H to exclude doublets from the analysis.

### Senescence-associated β-galactosidase staining

β-galactosidase activity at suboptimal pH (6.0) was used as a marker to detect cellular senescence^42^. Cells were seeded in four-well chamber slides at a density of 8000-10,000 cells. On the following day, the media was changed to low serum media (1.5%) and cells were treated with EPI-002 or ATRA. Three days later, the media was removed and cells were fixed with 2% formaldehyde/0.2% glutaraldehyde for 10 min at room temperature, washed with PBS, and then incubated with freshly prepared β-Gal staining solution (Cell Signaling) titrated to pH 6.0. Staining was optimal after incubating with the β-Gal solution at 37 °C for 16–18 h. After staining, the slides were washed twice with PBS, once with absolute methanol, and then mounted with 50% glycerol/PBS. The percentage of β-galactosidase-positive cells were counted with ImageJ using the Cell Counter plugin. A total of nine images containing 100–300 cells were scored to determine the percentages of positive cells.

### Animal studies

All experiments involving animals conform to the relevant regulatory and ethical standards and were approved by the University of British Columbia Animal Care Committee (A18-0077). Metacam (1 mg/kg, 0.05 mL/10 g of body weight) was administered subcutaneously prior to any surgery. Animals were anesthetized with isoflurane and euthanized by CO_2_. Six–eight-week-old male (NOD-*scid* IL2Rgamma^null^) mice were maintained in the Animal Care Facility at the British Columbia Cancer Research Centre. Mice were castrated 2 weeks before they were subcutaneously inoculated in the right flank with two million LN95-D3 cells in a 1:1 volume of matrigel (Corning Discovery Labware, Corning, NY). Tumor volume was measured twice a week by digital calipers and calculated by the formula: length × width × height × 0.5236. ATRA (5 mg, 21-day release) or a matching placebo pellet purchased from Innovative Research of America (Sarasota, FL) was subcutaneously implanted in the left side of the neck of mice 3 weeks after inoculating the cells or when tumor volume was about 33 mm^3^. Starting 3 days after the implants, animals were treated daily by oral gavage with 30 mg/kg body weight of EPI-7170 or vehicle (3% DMSO/1.5% Tween-80/1% CMC). Tumors were excised 1 day after the last dose and prepared for western blot analysis and immunohistochemistry.

### Immunohistochemistry

Sections were cut from formalin-fixed, paraffin-embedded tumor tissue at a thickness of 5 µm, deparaffinized in xylene, and then rehydrated in alcohols and distilled water. Endogenous peroxidase was blocked by incubating with a 3% hydrogen peroxide solution for 5 min. Sections were incubated overnight at 4 °C with antibodies recognizing the Ki-67 antigen (MIB-1; Dako Omnis) or antibodies specific for the AR N-terminus (PG-21; Millipore) at a dilution of 1:50. PSA was detected using a monoclonal mouse anti-human PSA antibody concentrate (ER-PR8; Dako). A negative control without the primary antibody was included for each sample on the same slide. Antigen was detected with 3,3-diaminobenzidine (DAB) and hematoxylin was used for counterstaining. Images were captured on an Axio Imager M2 microscope (Zeiss) at ×200 magnification. The percentage of Ki-67-positive cells was determined with ImageJ using the Cell Counter plugin. At least 1000 cells were scored from three images of representative tumors for each treatment group. The intensity of DAB staining for PSA was quantified by ImageJ using color deconvolution and measuring the mean gray value for the DAB image, where optical density = log(max intensity/mean intensity), and a max intensity of 255 for an 8-bit image.

### Quantification of serum PSA

Serum levels for total PSA were measured by a PSA ELISA kit (Anogen, Mississauga, ON) with a lower limit sensitivity of ~1 ng/mL. Whole blood was sampled from the tail vein on days indicated or by cardiac puncture immediately after euthanasia. The serum fraction was separated by centrifugation at 12,000 rpm for 3 min at room temperature and then stored at −20 °C until further analysis. Serum samples were diluted tenfold for the assay and were compared to calibration standards, according to the manufacturer’s protocol. The concentration of total PSA was determined by a VersaMax ELISA microplate reader by measuring absorbance at 450 nm with a reference wavelength of 650 nm.

### Statistics and reproducibility

Synergistic interactions between compounds were evaluated according to the Bliss independent model for a predicted additive effect,$F_{\rm{a}} + F_{\rm{b}} - \left( {F_{\rm{a}} \times F_{\rm{b}}} \right)$, where *F*_a_ and *F*_b_ are the fractional responses for treatment A and B at a given dose^38^. The CI was determined by the equation $\left[ {F_{\rm{a}} + F_{\rm{b}} - \left( {F_{\rm{a}} \times F_{\rm{b}}} \right)} \right]/F_{\rm{ab}}$ where *F*_ab_ represents the fractional response for the combination of A and B. Drug combinations were considered to be synergistic if CI < 1, additive if CI = 0, and antagonistic if CI > 1. Statistic differences were determined by GraphPad Prism 8 (San Diego, CA) by analysis of variance (ANOVA) unless stated otherwise. We used an alpha level of 0.05 for all statistical tests, where *P < 0.05, **P < 0.01, ***P < 0.001; ns not significant. Experiments were repeated with independent biological replicates at least three times.

### Reporting summary

Further information on research design is available in the Nature Research Reporting Summary linked to this article.

## Supplementary information

Peer Review File

Supplementary Information

Description of Additional Supplementary Files

Supplementary Data 1

Reporting Summary

## Data Availability

All data supporting the findings of this study are available within the paper and its supplementary information files. Source data for the figures can be found in Supplementary Data 1, and full western blot images are included in Supplementary Figs. 9–12.
