# Supplementary material for: Pin1 inhibition improves the efficacy of ralaniten compounds that bind to the N-terminal domain of androgen receptor
Source: Commun Biol. 2021 Mar 22;4:381. doi: 10.1038/s42003-021-01927-3 (PMC7985297; doi:10.1038/s42003-021-01927-3)
Supplement: Supplementary file 2 — Supplementary Information [file 42003_2021_1927_MOESM2_ESM.pdf]

Supplementary Figure 1

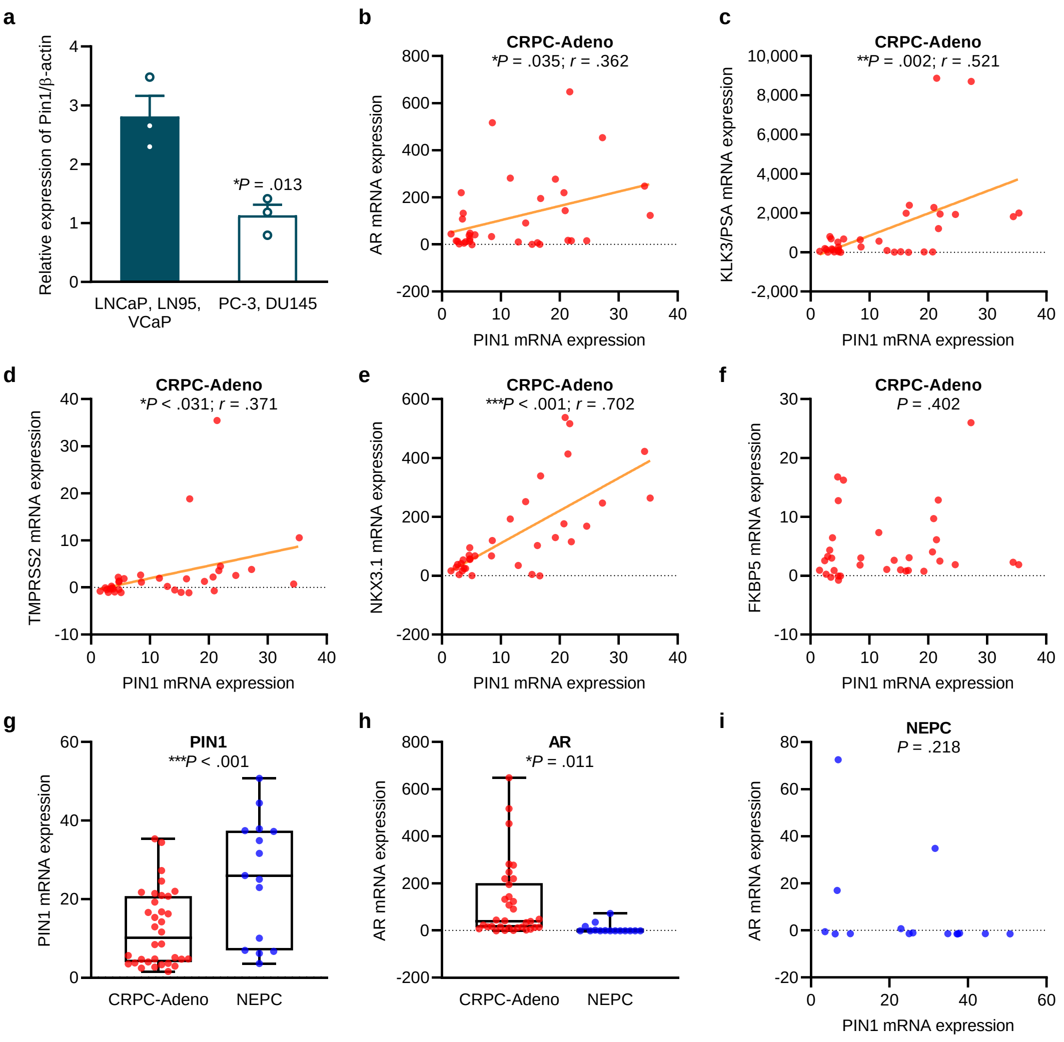

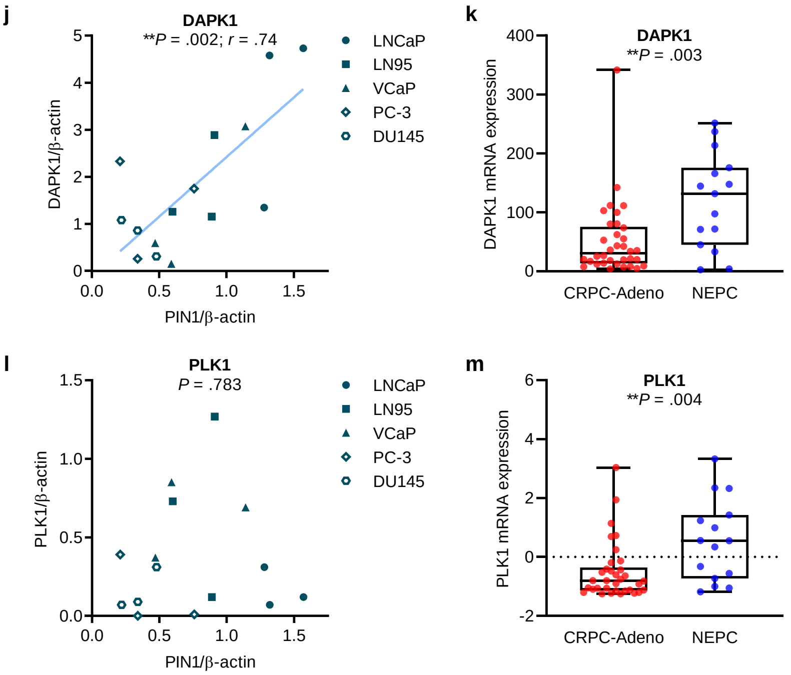

Supplementary Figure 1. Pin1 expression in clinical samples of prostate cancer and cell lines. (**a**) Graph summarizing the relative protein levels Pin1 of the cell lines based on AR expression. (**b**) Positive correlation between PIN1 and AR transcript levels from clinical samples of CRPC retrieved from the cBioPortal database. (**c-f**) Relationship between PIN1 levels and the expression of AR target genes in CRPC. (**g-h**) PIN1 and AR levels in CRPC and neuroendocrine prostate cancer. (**i**) Graph showing no correlation between PIN1 and AR levels in neuroendocrine prostate cancer. (**j-m**) Expression of DAPK1 and PLK1. Protein levels of (**j**) DAPK1 and (**l**) PLK1 in cell lines relative to Pin1 after normalizing to β-actin. Box plots compare the expression of (**k**) DAPK1 and (**m**) PLK1 mRNA in clinical samples. Statistical significance was determined by an independent two-tailed t-test.

Supplementary Figure 2

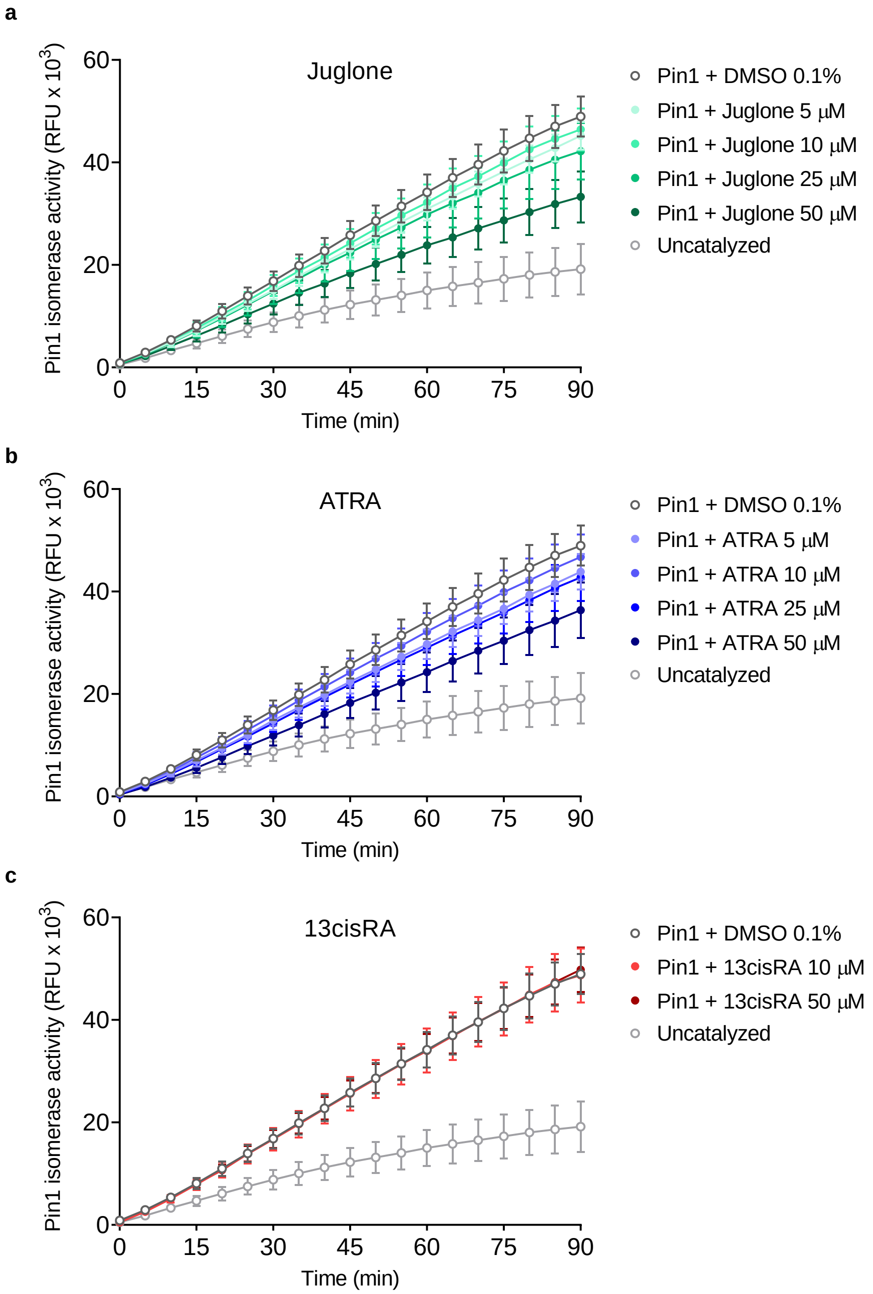

Supplementary Figure 2. Inhibition of Pin1 isomerase activity by pharmacological inhibitors. Fluorescent polarization-based assay showing the effects of (**a**) juglone, (**b**), ATRA, and (**c**) 13cisRA on the isomerase activity of Pin1. Each reaction contains 500 ng of recombinant human Pin1 protein incubated with an inhibitor and a fluorogenic substrate at 37°C. The uncatalyzed reaction represents the intrinsic *cis*/*trans* isomerization of the substrate without Pin1 included the reaction. Data points are the means ± s.e.m. from 3-4 separate experiments with the correlation performed in duplicate wells. RFU, relative fluorescent units.

Supplementary Figure 3

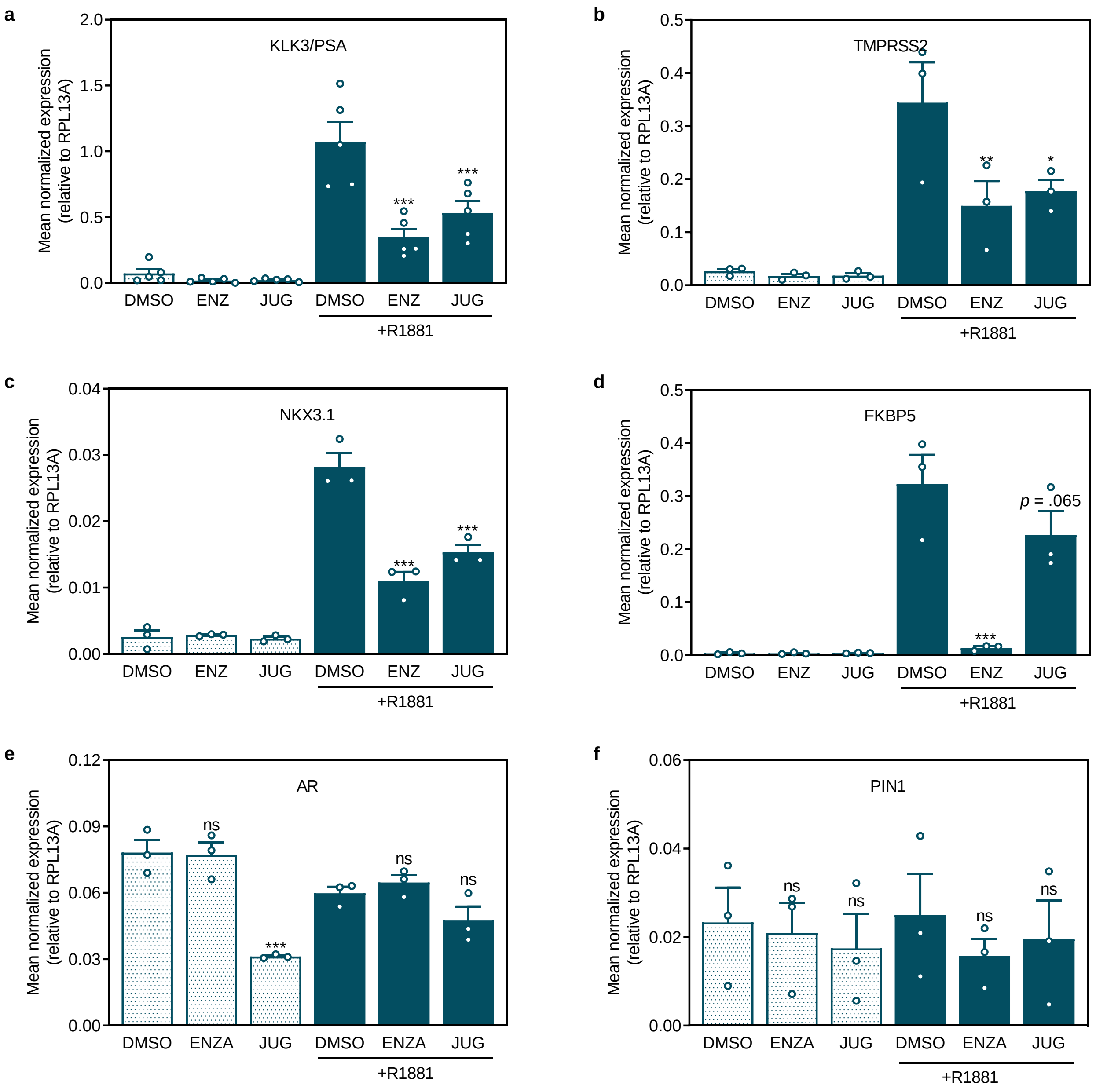

Supplementary Figure 3. Blocking isomerase activity of Pin1 inhibits the endogenous expression of androgen-regulated genes. Transcript levels from LNCaP cells after treating with vehicle (DMSO), enzalutamide (ENZ, 5 µM), juglone (JUG, 20 µM), and either ethanol or R1881 (1 nM) for 48 hours measured by quantitative real-time PCR. Data shown are the mean normalized expression (MNE) relative to the RLP13A housekeeping gene from 3-5 independent experiments. Statistical significance was determined by two-way ANOVA using Dunnett's multiple comparisons test. *P < 0.05, **P < 0.01, ***P < 0.001; ns, not significant.

Supplementary Figure 4

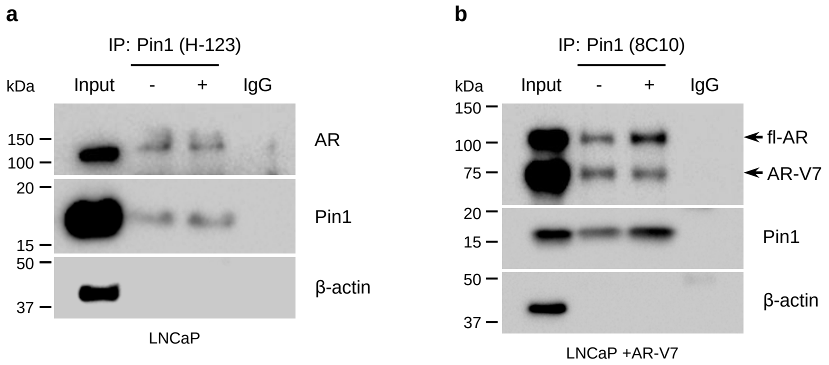

Supplementary Figure 4. Pin1 interacts with fl-AR and AR-V7. Co-immunoprecipitation assay showing interaction between (**a**) endogenous Pin1 and fl-AR from LNCaP whole cell lysates, and (**b**) Pin1 and AR-V7 from LNCaP cells with ectopically expressed AR-V7. Cells were incubated with 1 nM of R1881 (+) or vehicle (-) for 3 hours prior to harvesting lysates. Results shown are representative of 3-4 independent experiments. Immunoprecipitation with a non-specific IgG was included as a technical control, and β-actin was probed to show specificity of the assay.

Supplementary Figure 5

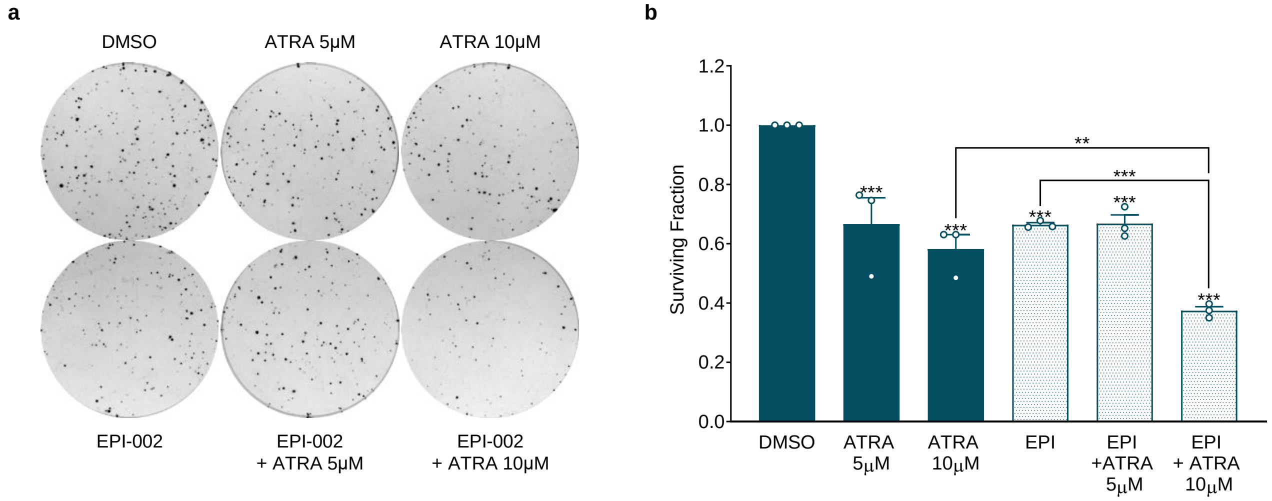

Supplementary Figure 5. Combination of ATRA and EPI inhibits colony formation of cells expressing full-length AR and AR-V7. (**a**) Representative images of colonies from LN95 cells after treatment with vehicle (DMSO), ATRA (10 µM), EPI-002 (25 µM), or combination of ATRA and EPI-002. After treating with compounds for 24 hours, the media was removed, and 500-1,000 cells were re-plated in 6-well plates. Colonies were fixed with 4% paraformaldehyde, stained with a 0.1% crystal violet solution, and then imaged. (**b**) The graph shows the surviving fraction of cells relative to the vehicle control, where foci were quantified using ImageJ software. Data represent the means ± s.e.m. from 3 separate experiments. Statistical significance was determined by one-way ANOVA using Holm-Sidak's multiple comparisons test. $**P < 0.01$, $***P < 0.001$.

Supplementary Figure 6

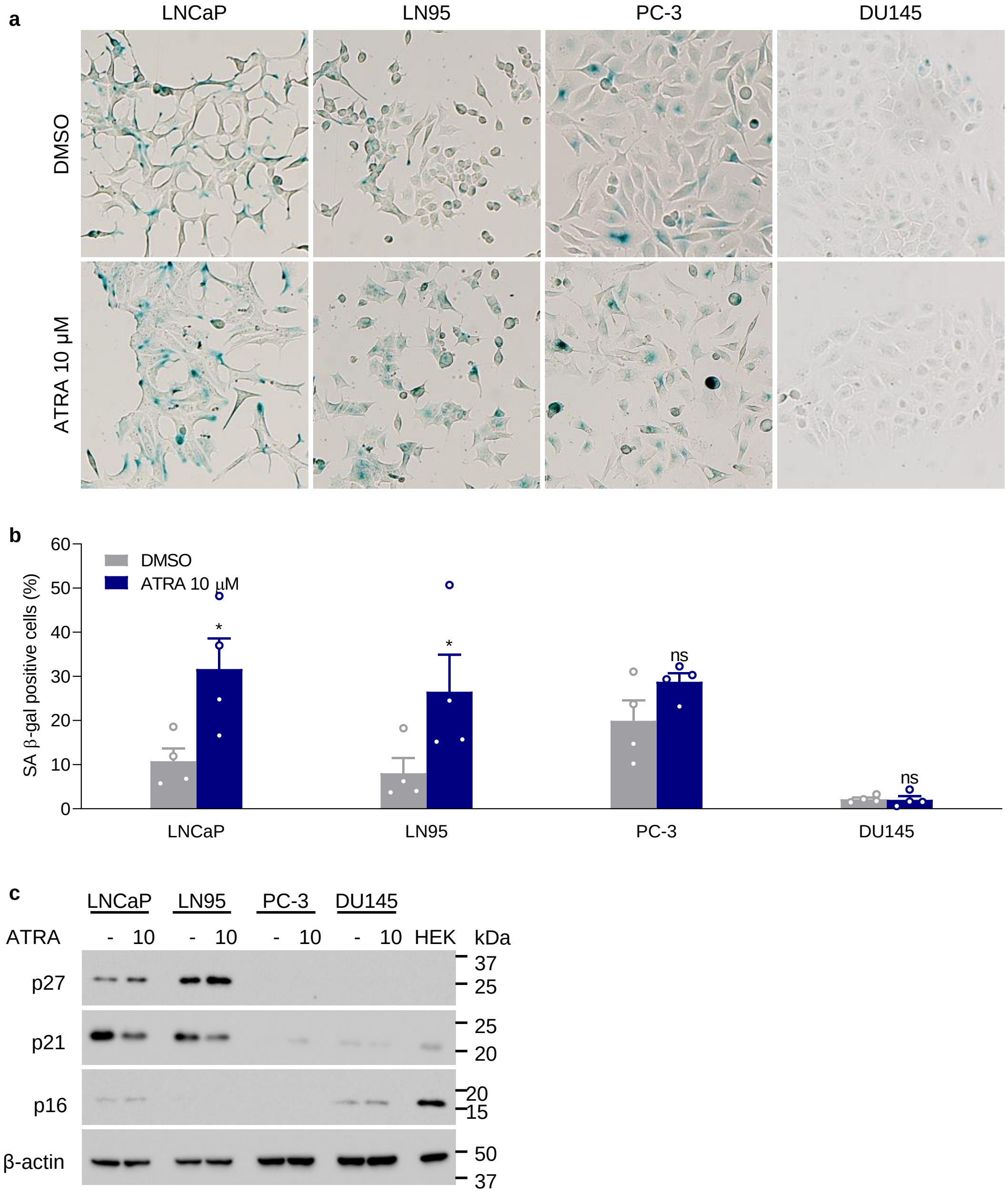

Supplementary Figure 6. Induction of senescence by ATRA on prostate cancer cell lines. (**a**) Staining of senescence-associated β-galactosidase (SA β-gal) activity in cell lines treated with vehicle (DMSO) or ATRA (10 µM) for 3 days. Original magnification ×100. (**b**) The graph shows the percentage of β-gal positive cells as means ± s.e.m. from 3 independent experiments, where at least 1,000 cells were scored for each group. (**c**) Representative Western blot showing the indicated cell cycle proteins. Statistical significance was determined by two-way ANOVA using Holm-Sidak's multiple comparisons test. *P < 0.05; ns, not significant.

Supplementary Figure 7

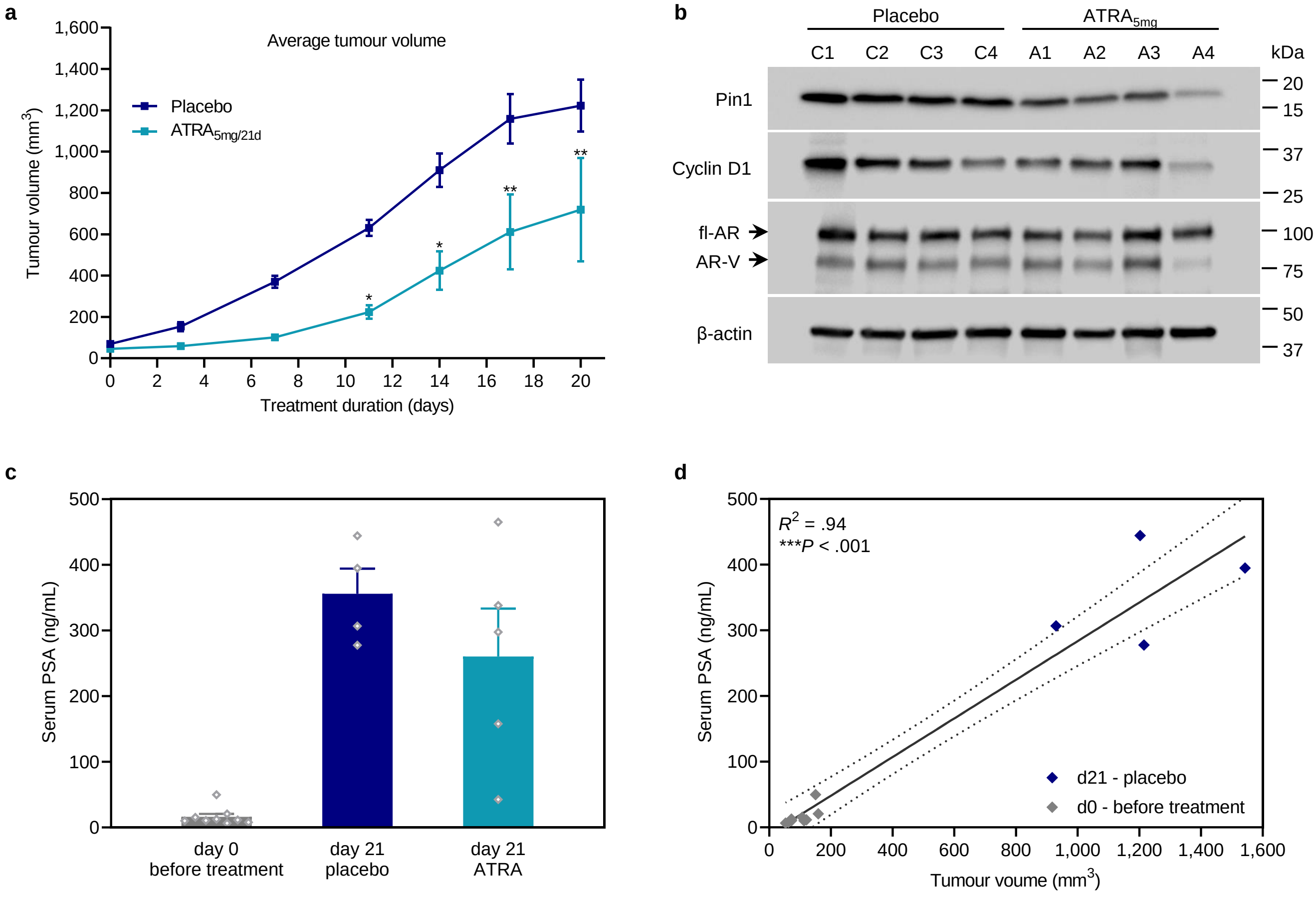

Supplementary Figure 7. Growth of LN95-D3 xenografts and in 10-week old castrated NSG mice bearing a subcutaneous 21-day release placebo or ATRA (5 mg) pellet. (**a**) Graph showing the average tumor volume from treatment plotted over time, where pellets were implanted on day 0. Error bars represent the mean ± s.e.m. Statistical significance was determined by mixed-effects analysis with Holm-Sidak's multiple comparisons test. *$P < 0.05$, **$P < 0.01$. (**b**) Western blot analysis of the xenograft tumors at the end of the study shows the on-target effect of ATRA with decreased levels of Pin1 and its target cyclin D1. (**c**) Serum PSA concentrations were determined from whole blood collected on day 0 (before initiating treatment) and day 21 from animals bearing placebo or ATRA (5 mg) pellets. Error bars represent the mean ± s.e.m. (**d**) Scatterplot graph shows a positive correlation between serum PSA and tumor volume, where the line of best fit is shown with 95% confidence bands.

Supplementary Figure 8

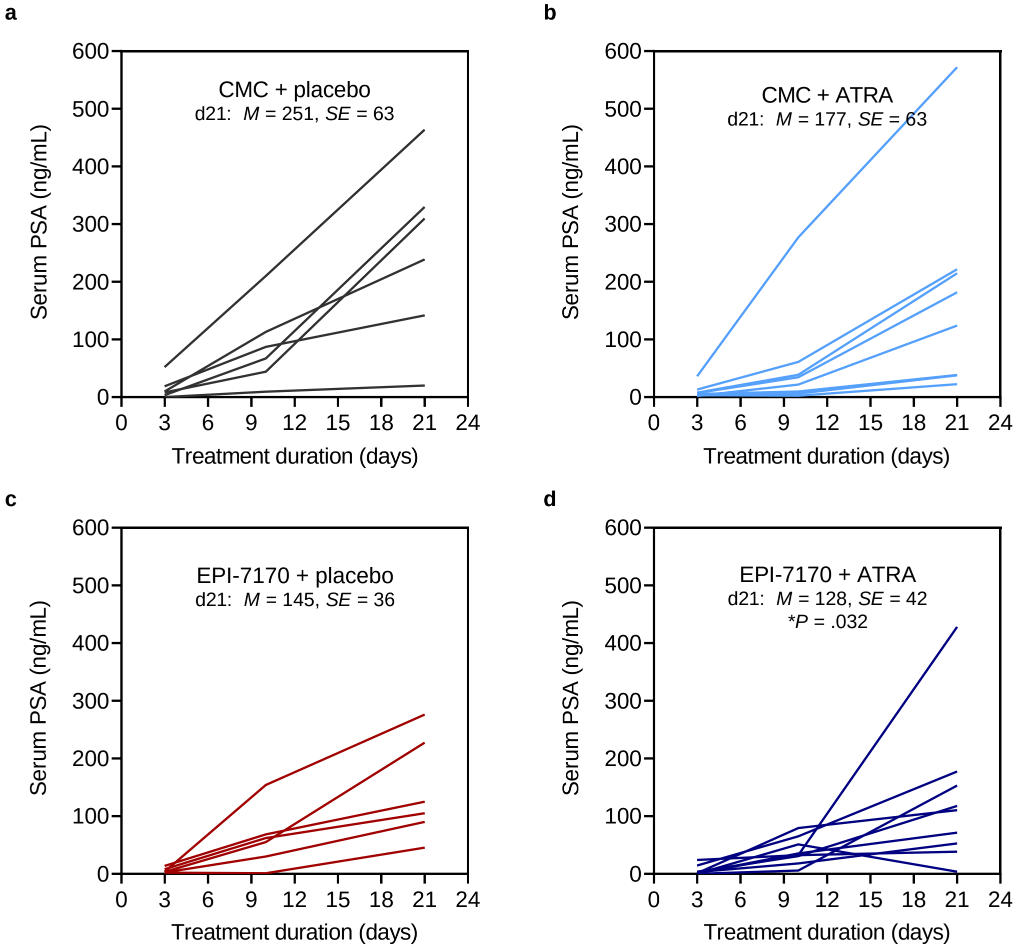

Supplementary Figure 8. Combination of ATRA and EPI reduces the expression of serum PSA from castrated mice bearing LN95-D3 xenografts. Levels of serum PSA were determined from whole blood collected on day 3, 10, and 21 from animals treated with (**a**) vehicle, (**b**) ATRA (5 mg), (**c**) EPI-7170 (30 mg/kg/d), or (**d**) combination. Data shown represents the PSA values from separate animals. Statistical significance was determined by two-way ANOVA using Dunnett's multiple comparisons test.

Supplementary Figure 9

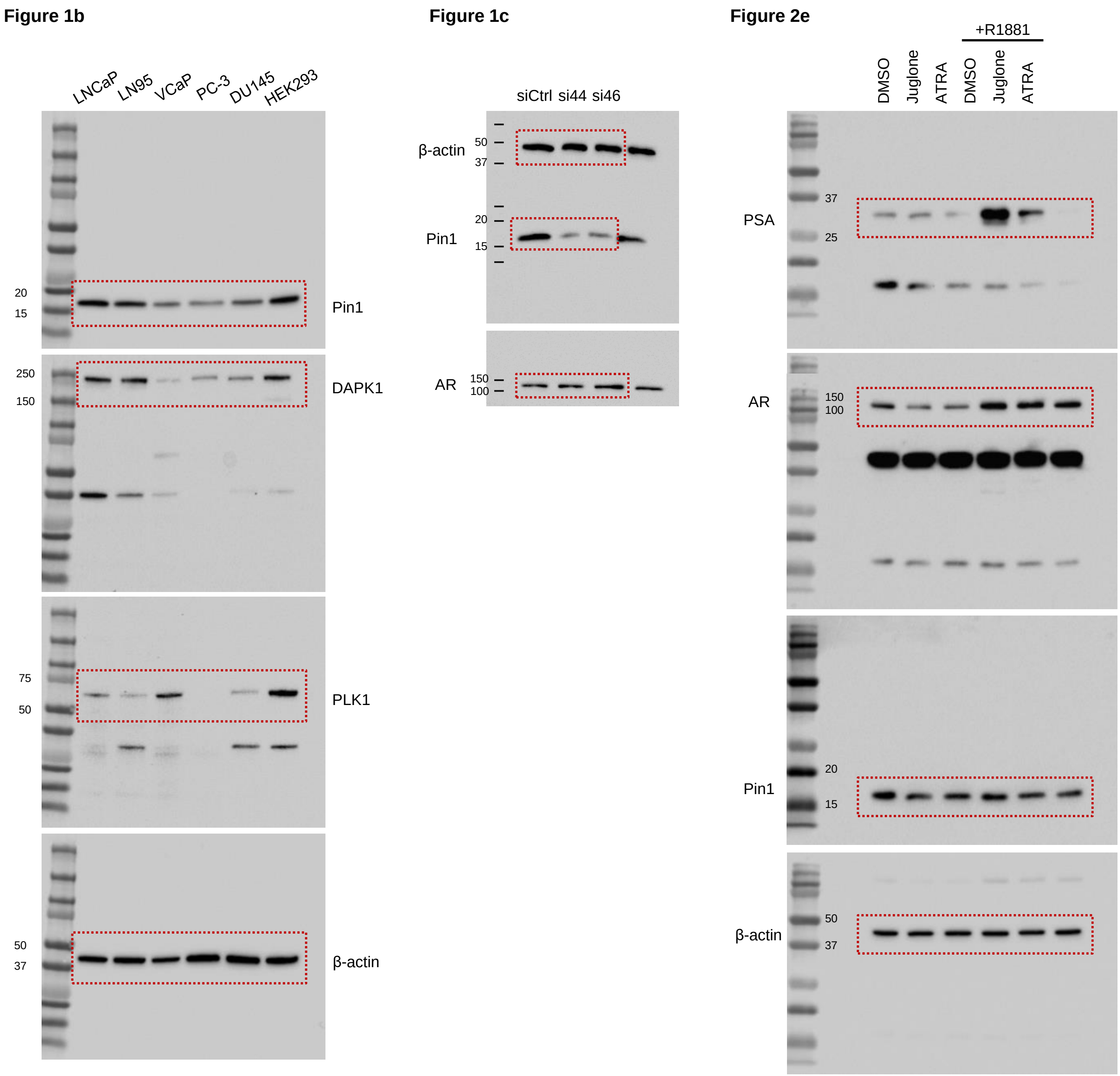

Supplementary Figure 9. Full Western blot images corresponding to Figures 1b, 1c, and 2e.

Supplementary Figure 10

Figure 3g

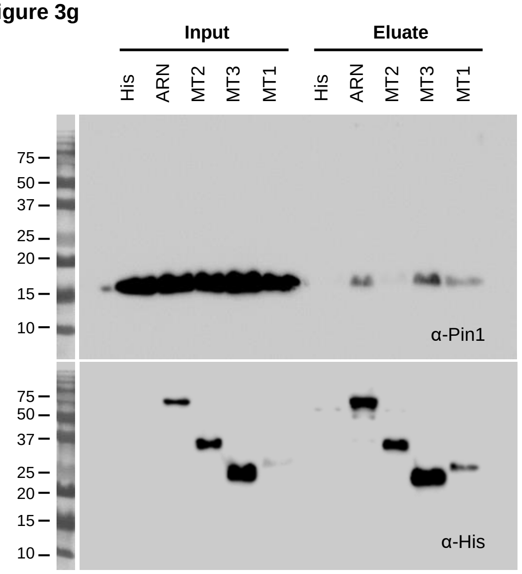

Figure 3h

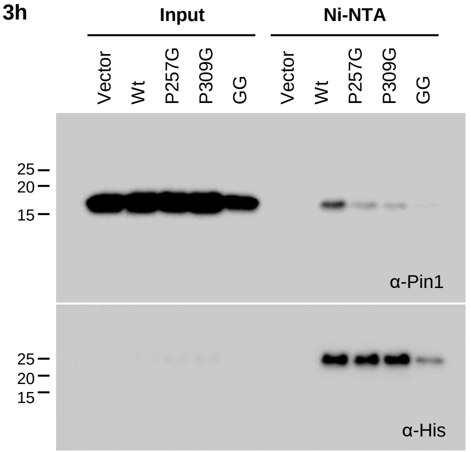

Figure 3i

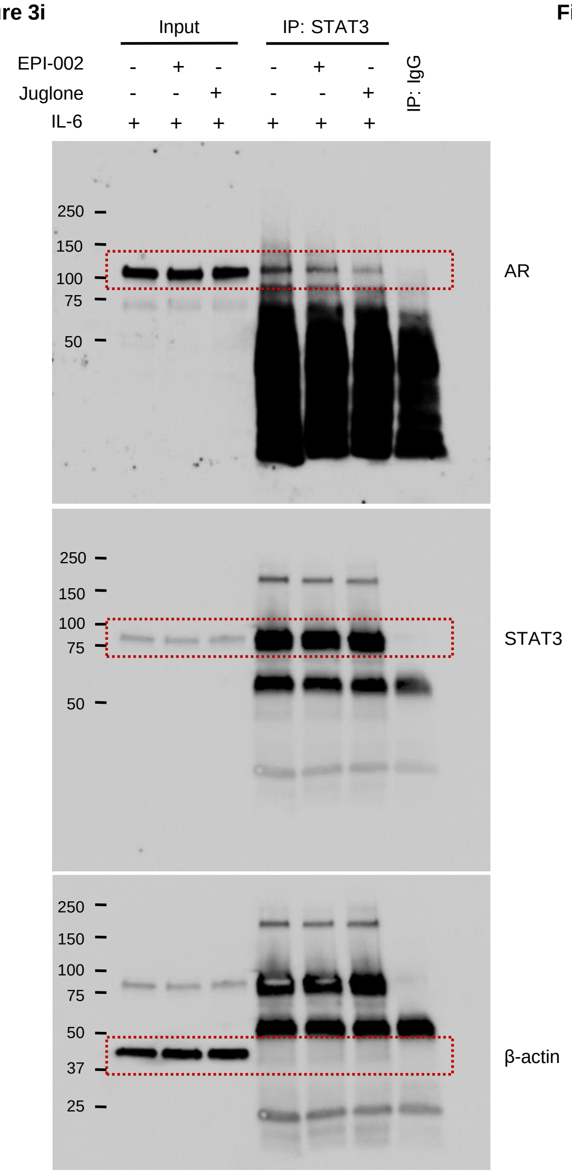

Figure 3j

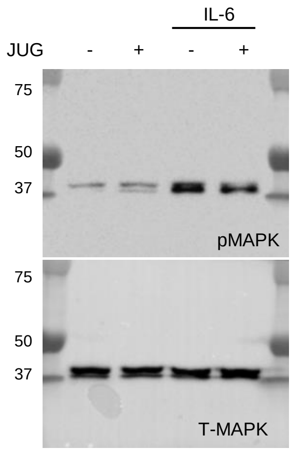

Figure 3k

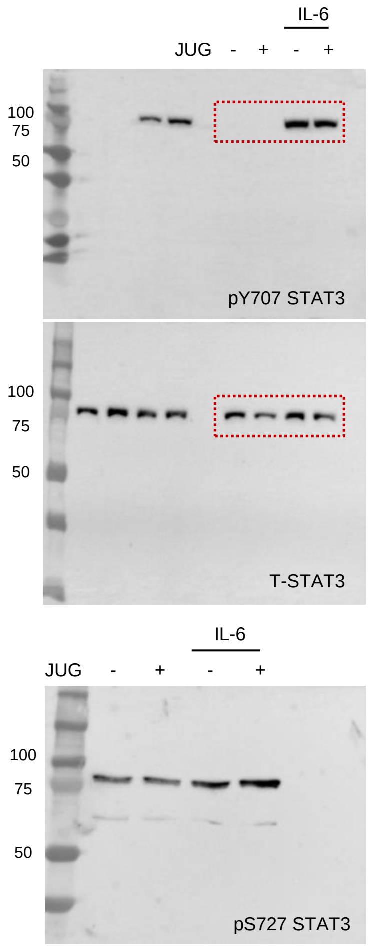

Supplementary Figure 10. Full Western blot images corresponding to Figures 3g-k.

Supplementary Figure 10

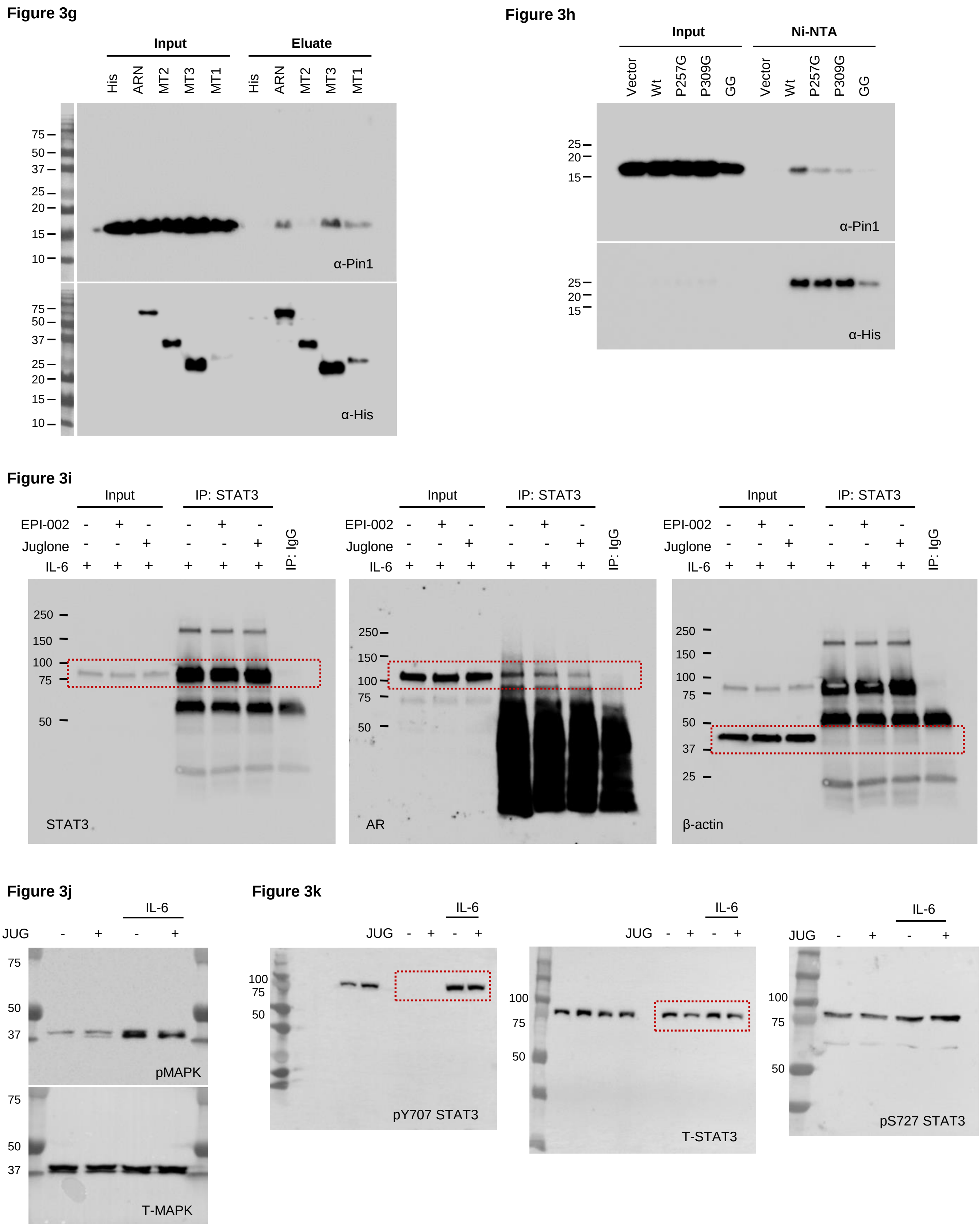

Supplementary Figure 10. Full Western blot images corresponding to Figures 3g-k.

Supplementary Figure 11

Figure 5c

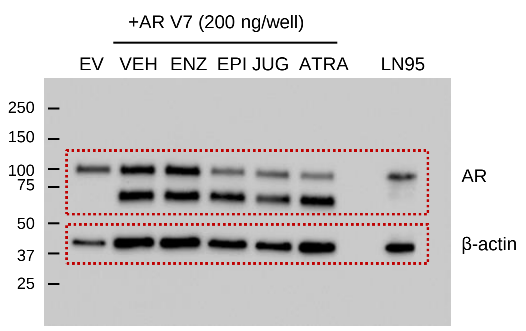

Figure 5d

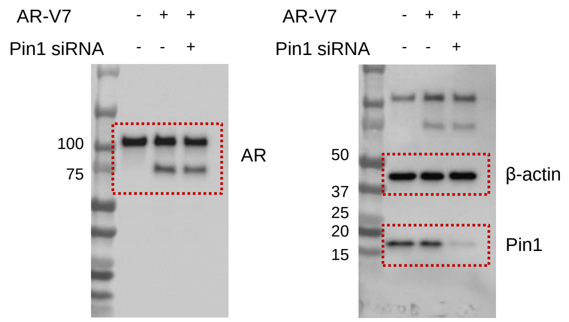

Figure 6a

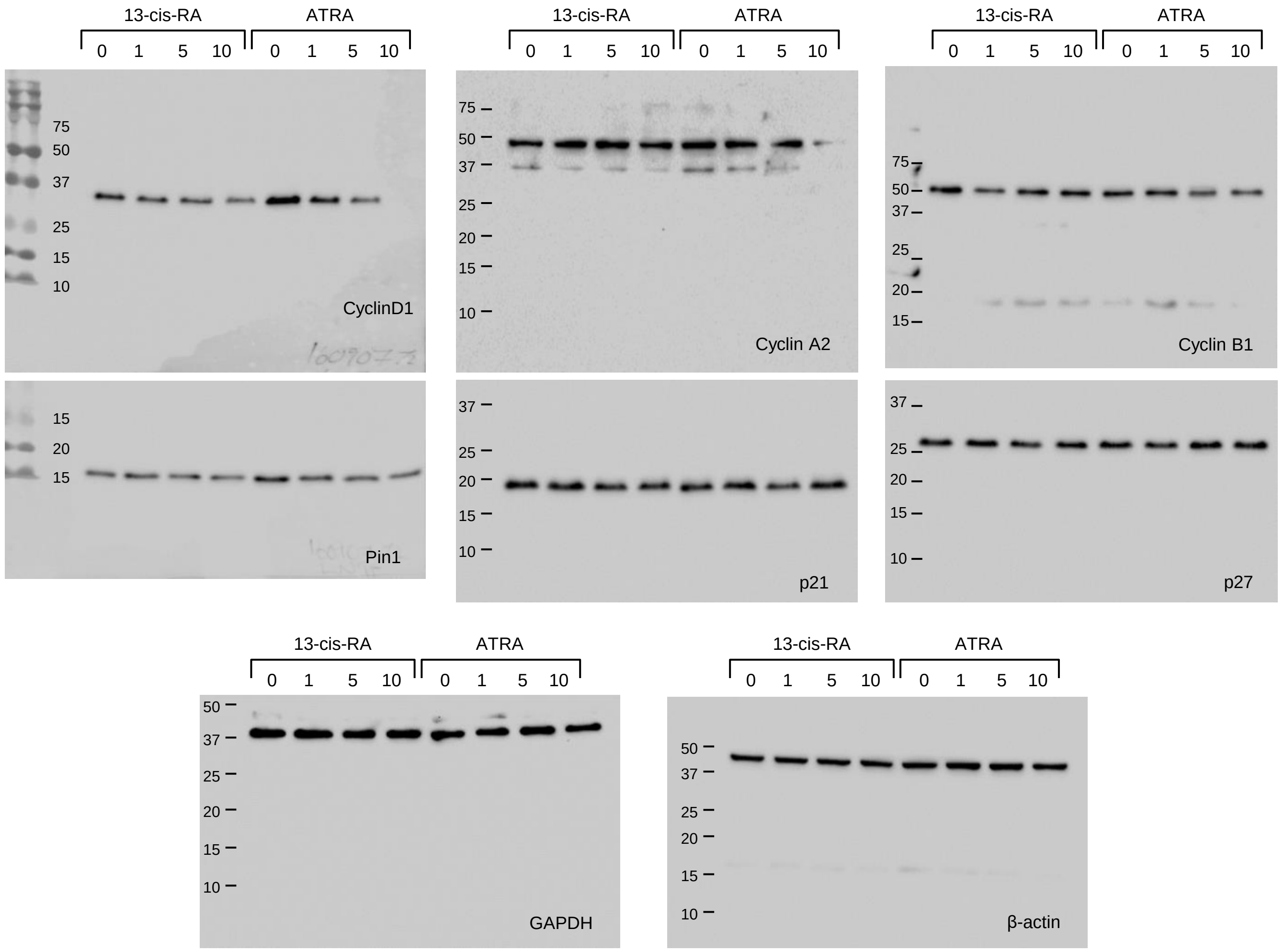

Supplementary Figure 11. Full Western blot images corresponding to Figures 5c, 5d, and 6a.

Supplementary Figure 12

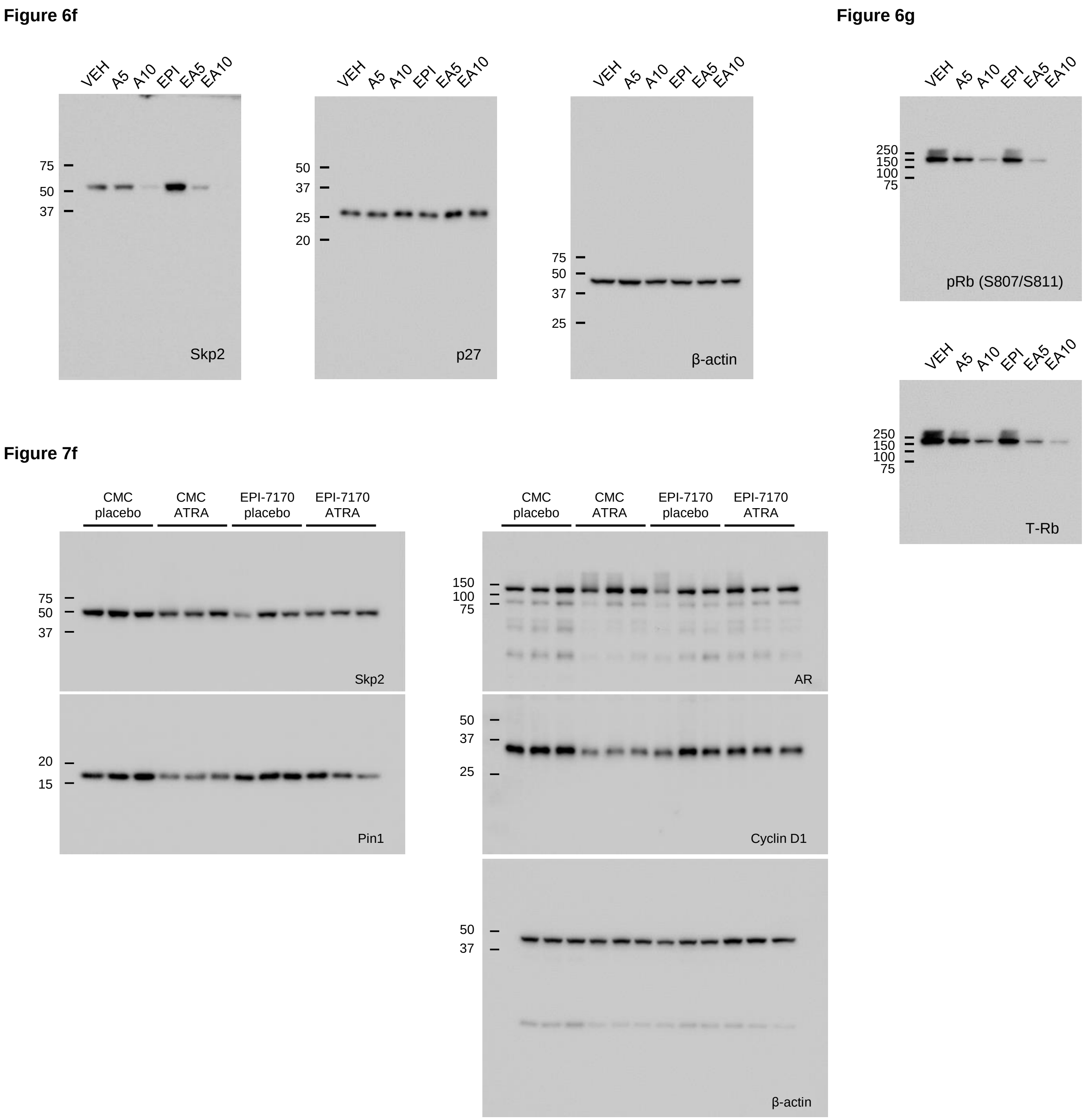

Supplementary Figure 12. Full Western blot images corresponding to Figures 6f, 6g, and 7f.
