# Supplementary material for: Pin1 inhibition improves the efficacy of ralaniten compounds that bind to the N-terminal domain of androgen receptor
Source: Commun Biol. 2021 Mar 22;4:381. doi: 10.1038/s42003-021-01927-3 (PMC7985297; doi:10.1038/s42003-021-01927-3)
Supplement: Supplementary file 3 — Description of Additional Supplementary Files [file 42003_2021_1927_MOESM3_ESM.pdf]

Description of Additional Supplementary Files

File name: Supplemental Data 1

Description: Source data behind the graphs in the paper.
