# Supplementary material for: Pin1 inhibition improves the efficacy of ralaniten compounds that bind to the N-terminal domain of androgen receptor
Source: Commun Biol. 2021 Mar 22;4:381. doi: 10.1038/s42003-021-01927-3 (PMC7985297; doi:10.1038/s42003-021-01927-3)
Supplement: Supplementary file 5 — Reporting Summary [file 42003_2021_1927_MOESM5_ESM.pdf]

nature research

Corresponding author(s): Marianne D Sadar

Last updated by author(s): Feb 14, 2020

Reporting Summary

Nature Research wishes to improve the reproducibility of the work that we publish. This form provides structure for consistency and transparency in reporting. For further information on Nature Research policies, see our Editorial Policies and the Editorial Policy Checklist.

Statistics

For all statistical analyses, confirm that the following items are present in the figure legend, table legend, main text, or Methods section.

n/a	Confirmed	
☐	☒	The exact sample size (*n*) for each experimental group/condition, given as a discrete number and unit of measurement
☐	☒	A statement on whether measurements were taken from distinct samples or whether the same sample was measured repeatedly
☐	☒	The statistical test(s) used AND whether they are one- or two-sided<br>*Only common tests should be described solely by name; describe more complex techniques in the Methods section.*
☐	☒	A description of all covariates tested
☐	☒	A description of any assumptions or corrections, such as tests of normality and adjustment for multiple comparisons
☐	☒	A full description of the statistical parameters including central tendency (e.g. means) or other basic estimates (e.g. regression coefficient) AND variation (e.g. standard deviation) or associated estimates of uncertainty (e.g. confidence intervals)
☐	☒	For null hypothesis testing, the test statistic (e.g. *F*, *t*, *r*) with confidence intervals, effect sizes, degrees of freedom and *P* value noted<br>*Give P values as exact values whenever suitable.*
☒	☐	For Bayesian analysis, information on the choice of priors and Markov chain Monte Carlo settings
☒	☐	For hierarchical and complex designs, identification of the appropriate level for tests and full reporting of outcomes
☐	☒	Estimates of effect sizes (e.g. Cohen's *d*, Pearson's *r*), indicating how they were calculated

Our web collection on statistics for biologists contains articles on many of the points above.

Software and code

Policy information about availability of computer code

Data collection	Software used to acquire images for Western blotting and microscopy are commercially available and described in the methods section.
Data analysis	ImageJ, Graphpad Prism, and FlowJo software were used for data analysis and the details are provided in the methods section.

For manuscripts utilizing custom algorithms or software that are central to the research but not yet described in published literature, software must be made available to editors and reviewers. We strongly encourage code deposition in a community repository (e.g. GitHub). See the Nature Research guidelines for submitting code & software for further information.

Data

Policy information about availability of data

All manuscripts must include a data availability statement. This statement should provide the following information, where applicable:

- Accession codes, unique identifiers, or web links for publicly available datasets
- A list of figures that have associated raw data
- A description of any restrictions on data availability

All data supporting the findings of this study are available within the paper and its supplementary information files.

Field-specific reporting

Life sciences study design

All studies must disclose on these points even when the disclosure is negative.

Sample size	Sample sizes and the number of independent experiments are disclosed in the figure legends. A simple power calculation was performed to predetermine the number of animals for the study, which was based on variability from pilot studies and experience with other animal models used in our lab.
Data exclusions	No data were excluded from the analyses.
Replication	All conclusions in our study were obtained from at least three biological replicates.
Randomization	Cell lines were passaged evenly into cell culture plates and dishes for treatment with test compounds. Animals were assigned to treatment groups when tumors reached a predetermined tumor volume.
Blinding	Investigators were not blinded to the nature of the sample during data collection and analysis.

Reporting for specific materials, systems and methods

We require information from authors about some types of materials, experimental systems and methods used in many studies. Here, indicate whether each material, system or method listed is relevant to your study. If you are not sure if a list item applies to your research, read the appropriate section before selecting a response.

Materials & experimental systems

n/a	Involved in the study	
☐	☒	Antibodies
☐	☒	Eukaryotic cell lines
☒	☐	Palaeontology and archaeology
☐	☒	Animals and other organisms
☒	☐	Human research participants
☒	☐	Clinical data
☒	☐	Dual use research of concern

Methods

n/a	Involved in the study	
☒	☐	ChIP-seq
☐	☒	Flow cytometry
☒	☐	MRI-based neuroimaging

Antibodies

Antibodies used	Primary antibodies against various proteins were from the following sources, mouse monoclonal antibodies: Pin1 (8C10), Cyclin D1 (DCS-6) from Santa Cruz Biotechnology; STAT3 (124H6), Cyclin A2 (BF683), Rb (4H1) from Cell Signaling Technology; GAPDH (6C5) from Invitrogen; β-actin (A5441), polyhistidine (HIS-1) from Sigma-Aldrich; rabbit monoclonal: DAPK1 (#3008), PLK1 (208G4), Cyclin B1 (D5C10), p16 INK4a (D3W8G), p21 Waf1/Cip1 (12D1), p27 Kip1 (D69C12), PSA/KLK3 (D11E1), phospho-Rb (Ser807/811; D20B12) from Cell Signaling Technology; rabbit polyclonal: Androgen Receptor (N-20) from Santa Cruz Biotechnology; phospho-STAT3 (Tyr705; D3A7), p44/42 MAPK (9102), phospho-p44/42 MAPK (Thr202/Tyr204), Skp2 (D3G5) from Cell Signaling Technology.
Validation	Validation was based on previous uses by our colleagues and by our lab, showing an expected size and labeling pattern.

Eukaryotic cell lines

Policy information about cell lines

Cell line source(s)	Cell lines were obtained from the following sources: LNCaP cells from Dr. Leland Chung (Cedars-Sinai Medical Centre, Los Angles, California); LNCaP95 (LN95) cells from Dr. Stephen Plymate (University of Washington, Seattle, Washington); VCaP cells and PC-3 cells from the American Type Culture Collection (Manassas, Virginia); and DU145 cells from Dr. Victor Ling (British Columbia Cancer Agency, Vancouver, British Columbia).
Authentication	LNCaP, VCaP, PC-3, and DU145 cells were authenticated by short tandem repeat analysis by DDC Medical (Fisher Scientific, Ottawa, Ontario).
Mycoplasma contamination	LNCaP, VCaP, PC-3, and DU145 cells were routinely tested to ensure they were mycoplasma-free by DDC Medical (Fisher Scientific, Ottawa, Ontario).
Commonly misidentified lines (See ICLAC register)	None of the cell lines used in this study are listed in the current version (v10) of the ICLAC register released on March 25, 2020.

Animals and other organisms

Policy information about studies involving animals; ARRIVE guidelines recommended for reporting animal research

Laboratory animals	NSG mice (NOD-scid IL2Rgammanull), male, 6-8 weeks old.
Wild animals	The study did not involve the use of wild animals.
Field-collected samples	The study did not involve the use of field-collected samples.
Ethics oversight	All experiments involving animals conform to the relevant regulatory and ethical standards and were approved by the University of British Columbia Animal Care Committee (A18-0077).

Note that full information on the approval of the study protocol must also be provided in the manuscript.

Flow Cytometry

Plots

Confirm that:

- [x] The axis labels state the marker and fluorochrome used (e.g. CD4-FITC).
- [x] The axis scales are clearly visible. Include numbers along axes only for bottom left plot of group (a 'group' is an analysis of identical markers).
- [x] All plots are contour plots with outliers or pseudocolor plots.
- [x] A numerical value for number of cells or percentage (with statistics) is provided.

Methodology

Sample preparation	Details for sample preparation and processing (fixation and labeling) are described in the methods section
Instrument	The data were collected with a BD FACSCalibur flow cytometer.
Software	The data were collected using CellQuest Pro and then analyzed by FlowJo V10 software.
Cell population abundance	Cell population abundance is reported as percentages in the graphs.
Gating strategy	Standard gating was performed on the samples to identify the cell population (FSC x SSC) and for doublet discrimination (FL3-W x FL3-H).

- [ ] Tick this box to confirm that a figure exemplifying the gating strategy is provided in the Supplementary Information.
