# Supplementary material for: Pin1 inhibition improves the efficacy of ralaniten compounds that bind to the N-terminal domain of androgen receptor
Source: Commun Biol. 2021 Mar 22;4:381. doi: 10.1038/s42003-021-01927-3 (PMC7985297; doi:10.1038/s42003-021-01927-3)
Supplement: Supplementary file 1 — Peer Review File [file 42003_2021_1927_MOESM1_ESM.pdf]

Reviewers' comments:

Reviewer #1 (Remarks to the Author):

In this paper, the authors studied the efficacy of a combination therapy of ralaniten, which has been tested clinically, and ATRA, which has already approved clinically for other indications, in treating castration-resistant prostate cancer. The article is well written and easy to understand and the results might have potential clinical implications. However there are some major concerns needed to addressed.

1: The authors claimed that Pin1 interacted with AR NTD. However there is little convincing data to demonstrate that Pin1 interacted with AR NTD in vitro and in vivo. They need to identify which Pin1 binds to which pSer/Thr-Pro motif and to mutate this phosphorylation motif disrupts the ability of Pin1 to regulate AR oncogenic functions in vitro and in vivo.

2. The current results that the high expression of Pin1 in androgen-sensitive cells such as LNCaP and the low expression of Pin1 in androgen-independent (AR non-reliant) highly malignant cells such as PC-3 and DU-145 (Fig. 1b, Supplementary Fig. 1a) are inconsistent with previous reports that high Pin1 levels are associated with recurrence.

3: In AR translocation assay (Fig. 2g), the results are not convincing. It is important to use subcellular fractionation to independent support the results of Pin1.

4: The authors showed similar effects of cis and trans RA such as in Fig. 2c, d. However, it has been shown that Pin1 binds to trans RA and cis RA needs to convert to trans RA so that cis RA is less active. Therefore, the authors need to do dose-dependent studies.

5: ATRA, Pin1 inhibitor, induces senescence in LNCaP, an androgen-sensitive cell, and suppresses its proliferation ability, but it does not work in AR-independent cells, PC-3 and DU-145. On the other hand, Juglone has an inhibitory effect on the proliferation of AR-independent cells (Fig. 4, Supplementary Fig. 5a, b). These results are not consistent with the idea that both Juglone and ATRA act through Pin1 inhibition. In fact, it is well known that Juglone has many Pin1-dependent function.

6: The therapeutic problem of castration-resistant prostate cancer (CRPC) is that hormone resistance is acquired in the process of cancer progression, and recurrence or metastasis. The authors demonstrate that combination therapy with ralaniten and ATRA inhibits the in vivo growth using subcutaneous xenograft model (Fig. 7b, d, e). Is combination therapy also effective against metastasis? The authors should do metastasis assay.

7: It is unclear how many mice were used in each treatment group in the CRPC xenografts model (Fig. 7 b, c). The number of tumors varies depending on the treatment group (Fig. 7 d, e). Is there any dropout during the course of treatment?

Minor points:

1. It would be better to describe DMSO, ATRA, EPI, etc. in columns like Fig. 6 C (Fig. 7 a).

2: In Fig.8 b-d, is the rightmost bar EPI/ATRA instead of EPI/placebo?

Reviewer #2 (Remarks to the Author):

In this study, the authors demonstrated the importance of PIN1 in regulating the transcriptional

activity of androgen receptor (AR) in castration-resistant prostate cancer (CRPC). Based on the pull-down assays, PIN1 was found to interact with the N-terminal domain (NTD) of AR. Inhibition of PIN1 expression by siRNA or its isomerase activity by ATRA and juglone resulted in a decrease of transcriptional activities of full-length AR and constitutively active AR splice variant (AR-Vs). In addition, inhibition of PIN1 activity by ATRA and juglone reduced cell proliferation of prostate cancer cells that are driven by full-length AR and AR-Vs. Combination treatment of PIN1 inhibitor ATRA and AR-NTD inhibitor ralaniten was synergistic in suppressing AR transcriptional activity and inducing cell cycle arrest. This combination treatment also showed superior anti-tumor effects against CRPC xenografts in vivo as compared with treatment with individual agent.

The manuscript was clearly written. Most of the experiments were carefully planned and controlled. I have the following comments which I hope the authors would address in the revised manuscript.

1. The authors demonstrated that PIN1 interacted with over-expressed AR NTD in LNCaP cells by pull-down assays(Fig. 3g). However, the authors did not confirm the interaction of endogenous full-length AR and AR-Vs with PIN1 in prostate cancer cells. Therefore, the author should perform co-immunoprecipitation assays to investigate whether endogenous full-length AR and AR-Vs interacts with PIN1 in LNCaP and LN95 cells, respectively.

2.In Fig. 3f, juglone has been found to inhibit transactivation activity of the AR 234-391 construct mutants (P257G and P309G) but not P257G/P309G double mutant. The authors suggested that these two putative PIN1 binding motifs (Ser256-Pro257 and Ser308-Pro309) located in the AR 234-391 region are essential to regulate AR transactivation. However, the authors did not demonstrate whether these two putative PIN1 binding motifs located in AR NTD are required for its interaction with PIN1. Therefore, the authors should perform the pull-down assay by using the cells transfected with various AR 234-391 construct mutants (P257G, P309G, and P257G/P309G).

3. Although the authors demonstrated that PIN1 is required for the transactivation activity of AR NTD, this effect may be mediated by the interaction between PIN1 and STAT3. Based on the study by Ueda et al., 2002, the interaction between STAT3 and AR NTD is involved in the increased transactivation activity of AR NTD. In addition, Lufei et al. (2007) have also demonstrated that PIN1 interacts with STAT3 to increase its binding to p300 for promoting its transcriptional activity. Therefore, PIN1 may first interact with STAT3 to promote its binding to AR NTD, leading to an increase of AR NTD transactivation activity. In Fig. 3h, the results showing the reduced amount of AR protein interacting with STAT3 by inhibiting PIN1 activity may support this notion. Therefore, the authors should determine whether PIN1 directly regulates the transactivation activity of AR NTD or or indirectly through STAT3 binding. For example, the authors may determine the effect of PIN1 in regulating transactivation activity of AR NTD in STAT3-depleted cells.

References:
Ueda et al., 2002: Activation of the androgen receptor N-terminal domain by interleukin-6 via MAPK and STAT3 signal transduction pathways

Lufei et al., 2007: Pin1 is required for the Ser727 phosphorylation-dependent Stat3 activity

4. As PIN1 expression promotes cell cycle progression and cell proliferation by regulating various oncogenic proteins e.g. cyclin D1 and p27, inhibition of cell cycle progression (Fig. 4 & 6c) and cell proliferation (Supplementary Fig. 4) by ATRA may not be mediated through PIN1-AR NTD axis. Therefore, the authors should determine whether the PIN1-induced transactivation activity of AR NTD contributes to cell proliferation.

5. The effects of PIN1 on AR were assessed using genetic knockdown or chemical inhibition. The authors should consider using PIN1 over-expression to demonstrate the positive regulation of AR transcription activity in prostate cancer cell lines.

6. The relationship between PIN1 expression and AR-dependency in prostatic cancer should be further validated in human samples.

Minor comments

1. The authors should describe how to determine PIN1 isomerase activity in the section ‘Methods’.

2. Fig. 1a: Please label S650-P in hinge AR region but not S560-P.

3. P.9:In 2nd paragraph, the figure showing ‘PIN1 interacted with the AR NTD’ should be in Fig. 3g but not Fig. 2g.

4. Fig. 6c: Please label clearly the concentrations of ATRA in columns 2 & 3 and state which row of BrdU results representing 24hr and 48hr incubation.

5. Fig. 7a: Please label clearly which graph is representing monotherapy or combination therapy of PIN1 and AR inhibitors.

6. Fig. 8b-d:The last column should be labeled as combination treatment of EPI with ATRA but not EPI with placebo.

Comments from Reviewer 1:

In this paper, the authors studied the efficacy of a combination therapy of ralaniten, which has been tested clinically, and ATRA, which has already approved clinically for other indications, in treating castration-resistant prostate cancer. The article is well written and easy to understand and the results might have potential clinical implications. However there are some major concerns needed to addressed.

Comment 1: *The authors claimed that Pin1 interacted with AR NTD. However there is little convincing data to demonstrate that Pin1 interacted with AR NTD in vitro and in vivo. They need to identify which Pin1 binds to which pSer/Thr-Pro motif and to mutate this phosphorylation motif disrupts the ability of Pin1 to regulate AR oncogenic functions in vitro and in vivo.*

Response: Evidence of interaction between Pin1 and the AR NTD is provided with pull-down assays shown in **Fig. 3g**. Site-directed mutagenesis experiments provided in **Fig. 3f** shows that mutation of the Ser-Pro257 or Ser-P309G motifs attenuated ARN transactivation. For controls, we have added pull-down assays showing that Pin1 does not interact with AR 234-391 of the AR NTD when both of these motifs have been mutated (**Fig. 3h**). Thus, our data show that Ser-Pro257 and Ser-P309G motifs are responsible for regulating AR NTD transactivation. Other Pin1 motifs are probably involved when AR is activated by androgen or stimulated by forskolin/cAMP signaling, which may have other oncogenic functions; however, the role of IL-6/STAT3 signaling in prostate cancer and its impact on AR NTD is well known. References include those below.

References:

Ueda, T., Mawji, N. R., Bruchovsky, N. & Sadar, M. D. Ligand-independent activation of the androgen receptor by interleukin-6 and the role of steroid receptor coactivator-1 in prostate cancer cells. *J. Biol. Chem.* **277**, 38087-38094, doi:10.1074/jbc.M203313200 (2002).

Malinowska, K. *et al.* Interleukin-6 stimulation of growth of prostate cancer in vitro and in vivo through activation of the androgen receptor. *Endocr. Relat. Cancer* **16**, 155-169, doi:10.1677/ERC-08-0174 (2009).

Comment 2: *The current results that the high expression of Pin1 in androgen-sensitive cells such as LNCaP and the low expression of Pin1 in androgen-independent (AR non-reliant) highly malignant cells such as PC-3 and DU-145 (Fig. 1b, Supplementary Fig. 1a) are inconsistent with previous reports that high Pin1 levels are associated with recurrence.*

Response: PC-3 and DU145 do not represent the same disease as AR-positive prostate cancer such as LNCaP and LN95 cells and those AR-deficient malignancies represented by DU145 and PC-3 are treated differently in the clinic. Data provided here suggest that Pin1 expression in prostate cancer is associated with transcriptionally active AR. These data are consistent with findings from Bao et al. 2004 which reported higher Pin1 expression in patients with untreated (androgen-sensitive) prostate cancer (~65%) than patients with hormone-refractory prostate cancer (~55%). Elevated Pin1 levels in patient biopsies are associated with an increased likelihood of developing PSA recurrence after radical prostatectomy compared to patients with low Pin1 levels (Sasaki et al. 2006), but there are no reports of increased Pin1 expression after recurrence or in AR-null prostate cancer cells compared to AR-positive prostate cancer cells.

References:

Bao, L. *et al.* Prevalent overexpression of prolyl isomerase Pin1 in human cancers. *Am. J. Pathol.* **164**, 1727-1737, doi:10.1016/S0002-9440(10)63731-5 (2004).

Sasaki, T. *et al.* An immunohistochemical scoring system of prolyl isomerase Pin1 for predicting relapse of prostate carcinoma after radical prostatectomy. *Pathol. Res. Pract.* **202**, 357-364, doi:10.1016/j.prp.2005.12.007 (2006).

Comment 3: *In AR translocation assay (Fig. 2g), the results are not convincing. It is important to use subcellular fractionation to independent support the results of Pin1.*

Response: The fluorescent microscopy data using YFP-AR included numerous controls that include a vehicle (DMSO), androgen (with and without), and antiandrogens (bicalutamide, enzalutamide), and yields data that are consistent with reported data from other groups such as Dr. Charles Sawyers. These AR translocation data in **Fig. 2d** shows the subcellular localization of YFP-AR in relationship to DAPI staining of the nucleus using microscopy. These data show differences that were quantified in **Fig. 2e**, where the nuclear to cytoplasmic ratio of YFP-AR was measured from at least 50 cells per treatment and three independent experiments. Since receiving this comment, we attempted subcellular fractionation experiments but were unable to resolve issues with methodology in order to obtain the expected results with these established controls thereby making the data unreliable. Presumably, the poor results with subcellular fractionation were due to issues based upon the known differences in AR binding to the chromatin amongst the different inhibitors and agonist. Weak interactions of AR with the chromatin may be lost during subcellular fractionation. For example, bicalutamide stimulates AR binding to chromatin, albeit weaker than with agonist, whereas enzalutamide does not stimulate AR binding to the chromatin (Dr. Steven Balk's work: Yuan X et al Oncogene 2014; Yu Z et al Clin Cancer Res 2014). These differences may be the reason why fluorescent microscopy rather than subcellular fraction were the method of choice in the publications examining AR nuclear translocation with AR inhibitors (e.g., Tran et al 2009 Science; Andersen et al Cancer Cell 2010; Clegg et al Cancer Res 2012; and more recent Wu et al The Prostate 2019). AR translocation data using a fluorescent fusion protein as used here can visualize AR localization in response to established controls and experimental inhibitors independently of AR binding affinity to chromatin.

References:

Yuan, X. *et al.* Androgen receptor functions in castration-resistant prostate cancer and mechanisms of resistance to new agents targeting the androgen axis. *Oncogene* **33**, 2815-2825, doi:10.1038/onc.2013.235 (2014).

Yu, Z. et al. Galeterone prevents androgen receptor binding to chromatin and enhances degradation of mutant androgen receptor. *Clin. Cancer Res.* **20**, 4075-85 (2014).

Comment 4: *The authors showed similar effects of cis and trans RA such as in Fig. 2c, d. However, it has been shown that Pin1 binds to trans RA and cis RA needs to convert to trans RA so that cis RA is less active. Therefore, the authors need to do dose-dependent studies.*

Response: Thank you for this suggestion. We have included the dose-response for 13cisRA in **Fig. 2a,b**. These data indeed show that 13cisRA has some activity but is less active than ATRA, likely from the conversion to ATRA, which is consistent with previous reports (**ref. 26**).

References:

Chen, H. & Juchau, M. R. Recombinant human glutathione S-transferases catalyse enzymic isomerization of 13-cis-retinoic acid to all-trans-retinoic acid in vitro. *Biochem. J.* **336 (Pt 1)**, 223-226, doi:10.1042/bj3360223 (1998).

Comment 5: *ATRA, Pin1 inhibitor, induces senescence in LNCaP, an androgen-sensitive cell, and suppresses its proliferation ability, but it does not work in AR-independent cells, PC-3 and DU-145. On the other hand, Juglone has an inhibitory effect on the proliferation of AR-independent cells (Fig. 4, Supplementary Fig. 5a, b). These results are not consistent with the idea that both Juglone and ATRA act through Pin1 inhibition. In fact, it is well known that Juglone has many Pin1-dependent function.*

Response: Juglone and ATRA inhibit Pin1 by distinct mechanisms of action. Juglone covalently binds to an active site cysteine in Pin1 while ATRA binds to active Pin1 and enhances the protein degradation of Pin1. Juglone's mechanism is relatively non-specific and off-target effects on other mitotic proteins are reported in Moore & Potter, 1998 (**ref. 34**). We state this in the manuscript on **page 9-10**: "Based on these findings, we focused on ATRA as a pharmaceutical inhibitor of Pin1 since its effects were consistent with knockdown of Pin1 whereas juglone has known cytotoxic effects on non-specific targets involved in mitosis[34]."

References:

Moore, J. D. & Potter, A. Pin1 inhibitors: Pitfalls, progress and cellular pharmacology. *Bioorg. Med. Chem. Lett.* **23**, 4283-4291, doi:10.1016/j.bmcl.2013.05.088 (2013).

Comment 6: *The therapeutic problem of castration-resistant prostate cancer (CRPC) is that hormone resistance is acquired in the process of cancer progression, and recurrence or metastasis. The authors demonstrate that combination therapy with ralaniten and ATRA inhibits the in vivo growth using subcutaneous xenograft model (Fig. 7b, d, e). Is combination therapy also effective against metastasis? The authors should do metastasis assay.*

Response: While it would be interesting to explore this aspect, our study is focused on the role of Pin1 in regulating the transcriptional activity of AR, as well as the potential for combination therapy of ralaniten and a Pin1 inhibitor to treat prostate cancer expressing AR including AR-Vs. The role of AR in the mechanism for metastases is not established and hence metastases are outside of the scope of the study. The FDA has permitted INDs for the clinical trial for ralaniten and its second-generation analogs for cohorts of metastatic CRPC patients that express the AR-regulated gene PSA and have failed abiraterone and/or enzalutamide. The LN95 cell line expresses AR and is enzalutamide-resistant but it has not been characterized for its metastatic behavior, and LNCaP cells are known to not metastasize in murine models. PC-3 and DU145 do metastasize, but these cell lines do not express functional AR, and therefore would not be suitable for testing combinations with ralaniten, which is the main focus of this manuscript.

Neuroendocrine and/or AR-deficient prostate cancer cells such as PC-3 and DU145 represent a different form of CRPC than AR-positive prostate cancer and are treated differently in the clinic.

Comment 7: *It is unclear how many mice were used in each treatment group in the CRPC xenografts model (Fig. 7 b, c). The number of tumors varies depending on the treatment group (Fig. 7 d, e). Is there any dropout during the course of treatment?*

Response: We intentionally included more animals in the treatment groups than the placebo control to account for the potential rare occurrence of adverse reactions to the ATRA pellets. We also needed to take into consideration the number of placebo or ATRA pellets available at the time. All tumors that were growing consistently before the start of treatment were included in the study such that there were no dropouts after treatment had been initiated.

Comments from Reviewer 2:

In this study, the authors demonstrated the importance of PIN1 in regulating the transcriptional activity of androgen receptor (AR) in castration-resistant prostate cancer (CRPC). Based on the pull-down assays, PIN1 was found to interact with the N-terminal domain (NTD) of AR. Inhibition of PIN1 expression by siRNA or its isomerase activity by ATRA and juglone resulted in a decrease of transcriptional activities of full-length AR and constitutively active AR splice variant (AR-Vs). In addition, inhibition of PIN1 activity by ATRA and juglone reduced cell proliferation of prostate cancer cells that are driven by full-length AR and AR-Vs. Combination treatment of PIN1 inhibitor ATRA and AR-NTD inhibitor ralaniten was synergistic in suppressing AR transcriptional activity and inducing cell cycle arrest. This combination treatment also showed superior anti-tumor effects against CRPC xenografts in vivo as compared with treatment with individual agent.

The manuscript was clearly written. Most of the experiments were carefully planned and controlled. I have the following comments which I hope the authors would address in the revised manuscript.

Comment 1: *The authors demonstrated that PIN1 interacted with over-expressed AR NTD in LNCaP cells by pull-down assays(Fig. 3g). However, the authors did not confirm the interaction of endogenous full-length AR and AR-Vs with PIN1 in prostate cancer cells. Therefore, the author should perform co-immunoprecipitation assays to investigate whether endogenous full-length AR and AR-Vs interacts with PIN1 in LNCaP and LN95 cells, respectively.*

Response: As suggested, co-immunoprecipitation assays with the endogenous AR is provided in **Supplementary Fig. 4**. These data reveal the interaction between endogenous fl-AR and Pin1 in LNCaP whole cell lysates, regardless of androgen treatment (**Supplementary Fig. 4a)**. Interaction between Pin1 and AR-V7 was also noted with ectopic expression of AR-V7 to levels that are approximately 1:1 with fl-AR (**Supplementary Fig. 4b)**, which is similar to reporter assays shown in **Fig. 5c**. These additional data are included on **page 8** of the manuscript.

Comment 2: *In Fig. 3f, juglone has been found to inhibit transactivation activity of the AR 234-391 construct mutants (P257G and P309G) but not P257G/P309G double mutant. The authors suggested that these two putative PIN1 binding motifs (Ser256-Pro257 and Ser308-Pro309) located in the AR 234-391 region are essential to regulate AR transactivation. However, the authors did not demonstrate whether these two putative PIN1 binding motifs located in AR NTD are required for its interaction with PIN1. Therefore, the authors should perform the pull-down assay by using the cells transfected with various AR 234-391 construct mutants (P257G, P309G, and P257G/P309G).*

Response: As suggested, these pull-down assays with AR 234-391 mutant constructs are now provided in **Fig. 3h** and are described on **page 8**. These data show that Pin1 does not interact with the double mutant, which is consistent with transactivation assays in **Fig. 3f**.

Comment 3: *Although the authors demonstrated that PIN1 is required for the transactivation activity of AR NTD, this effect may be mediated by the interaction between PIN1 and STAT3. Based on the study by Ueda et al., 2002, the interaction between STAT3 and AR NTD is involved in the increased transactivation activity of AR NTD. In addition, Lufei et al. (2007) have also demonstrated that PIN1 interacts with STAT3 to increase its binding to p300 for promoting its transcriptional activity. Therefore, PIN1 may first interact with STAT3 to promote its binding to AR NTD, leading to an increase of AR NTD transactivation activity. In Fig. 3h, the results showing the reduced amount of AR protein interacting with STAT3 by inhibiting PIN1 activity may support this notion. Therefore, the authors should determine whether PIN1 directly regulates the transactivation activity of AR NTD or or indirectly through STAT3 binding. For example, the authors may determine the effect of PIN1 in regulating transactivation activity of AR NTD in STAT3-depleted cells.*

Response: We attempted this using stable cell lines expressing dominant-negative STAT3 mutants, but as expected this did not work because functional STAT3 is required for the IL-6-induced STAT3 interaction with the AR NTD to induce transactivation of AR NTD (Ueda et al 2002). Thus the experiment is simply not feasible.

References:

Ueda, T., Mawji, N. R., Bruchovsky, N. & Sadar, M. D. Ligand-independent activation of the androgen receptor by interleukin-6 and the role of steroid receptor coactivator-1 in prostate cancer cells. *J. Biol. Chem.* **277**, 38087-38094, doi:10.1074/jbc.M203313200 (2002).

Lufei, C., Koh, T. H., Uchida, T. & Cao, X. Pin1 is required for the Ser727 phosphorylation-dependent Stat3 activity. *Oncogene* **26**, 7656-7664, doi:10.1038/sj.onc.1210567 (2007).

Comment 4: *As PIN1 expression promotes cell cycle progression and cell proliferation by regulating various oncogenic proteins e.g. cyclin D1 and p27, inhibition of cell cycle progression (Fig. 4 & 6c) and cell proliferation (Supplementary Fig. 4) by ATRA may not be mediated through PIN1-AR NTD axis. Therefore, the authors should determine whether the PIN1-induced transactivation activity of AR NTD contributes to cell proliferation.*

Response: To address this is challenging since overexpression of Pin1 was not feasible due to the already high expression of Pin1 in these models and knockdown of Pin1 or AR reduces the proliferation of prostate cancer cells. We do show that genetic and pharmaceutical inhibition of Pin1 by ATRA blocks

transactivation of the AR-NTD (Fig 3), which is consistent with ATRA decreasing AR-driven proliferation in Fig 4. So this query is addressed in these two figures.

Additionally, while we agree with your comment, demonstrating that ATRA's effect is mediated solely through the Pin1-AR NTD axis is challenging since either knockdown of Pin1 or AR reduces the proliferation of prostate cancer cells. Cyclin D1 is a validated Pin1 substrate, in which Pin1 enhances nuclear localization and protein stability of cyclin D1 via its Thr286-Pro motif (Liou et al., 2002). Moreover, Langenfeld et al. 1997 have reported that ATRA's effect on decreasing cyclin D1 levels occurs posttranslationally by a ubiquitin-dependent proteasome pathway with no effect on mRNA levels and therefore the decrease in cyclin D1 observed with ATRA cannot be justified by RA signaling and activation of RARs.

References:

Liou, Y. C. *et al.* Loss of Pin1 function in the mouse causes phenotypes resembling cyclin D1-null phenotypes. *Proc. Natl. Acad. Sci. U. S. A.* **99**, 1335-1340, doi:10.1073/pnas.032404099 (2002).

Langenfeld, J., Kiyokawa, H., Sekula, D., Boyle, J. & Dmitrovsky, E. Posttranslational regulation of cyclin D1 by retinoic acid: a chemoprevention mechanism. Proc. Natl. Acad. Sci. U. S. A. 94, 12070-12074, doi:10.1073/pnas.94.22.12070 (1997).

Comment 5: *The effects of PIN1 on AR were assessed using genetic knockdown or chemical inhibition. The authors should consider using PIN1 over-expression to demonstrate the positive regulation of AR transcription activity in prostate cancer cell lines.*

Response: In our hands, overexpression studies have not yielded meaningful outcomes, probably since prostate cancer cells already have elevated levels of Pin1 (Bao et al., 2004). Moreover, overexpression of Pin1 may also not localize correctly to regulate AR transcriptional activity.

References:

Bao, L. *et al.* Prevalent overexpression of prolyl isomerase Pin1 in human cancers. *Am. J. Pathol.* **164**, 1727-1737, doi:10.1016/S0002-9440(10)63731-5 (2004).

Comment 6: *The relationship between PIN1 expression and AR-dependency in prostatic cancer should be further validated in human samples.*

Response: There are already a number of publications on the levels of Pin1 in clinical samples of prostate cancer. In particular, in regard to AR-dependency in prostate cancer and Pin1 expression, there is an association of Pin1 with PSA recurrence which is an AR-regulated gene (Ayala et al. 2003; Sasaki et al. 2006). Ayala et al. state that "Pin1 expression had a significantly higher probability of recurrence than their counterparts with low Pin1 expression, as defined by a serum prostate-specific antigen level of ≥0.4 ng/ml on two consecutive occasions after radical prostatectomy. In addition, patients with high Pin1 expression had almost 4 times the risk of having earlier recurrence than those with low Pin1 expression; patients with a very high level had 8.1 times the risk of an earlier recurrence than a low Pin1 expresser." Sasaki et al. reported that "PCa patients assigned to the high Pin1 score group demonstrated PSA relapse more frequently than those assigned to the low Pin1 score group (p<0.0001)." These publications describe the relationship between elevated Pin1 expression and PSA relapse. The relationship between Pin1 expression and prostate cancer in comparison to other types of human cancers has also been previously reported by Bao et al. 2004. Here we refer to these publications and state "This enzyme is commonly

overexpressed in prostate cancer, and its expression in patient biopsies correlates with recurrence[10-12]." on **page 3**, and also "This *cis/trans* proline isomerase is commonly elevated in prostate cancer and associated with disease recurrence[10-12]" on **page 16**.

References:

Ayala, G. *et al.* The prolyl isomerase Pin1 is a novel prognostic marker in human prostate cancer. *Cancer Res.* **63**, 6244-6251 (2003).

Sasaki, T. *et al.* An immunohistochemical scoring system of prolyl isomerase Pin1 for predicting relapse of prostate carcinoma after radical prostatectomy. *Pathol. Res. Pract.* **202**, 357-364, doi:10.1016/j.prp.2005.12.007 (2006).

Bao, L. *et al.* Prevalent overexpression of prolyl isomerase Pin1 in human cancers. *Am. J. Pathol.* **164**, 1727-1737, doi:10.1016/S0002-9440(10)63731-5 (2004).

Additional Clarifications:

In addition to the comments mentioned above, all minor comments from both reviewers have been addressed in the revised manuscript. These changes include the addition of experimental details for the Pin1 isomerase activity assay in the Methods section and corrections to the labeling of the Figures, according to the reviewers' comments. We have also added *in vivo* PSA data (**Supplementary Figures 7 and 8**) to **page 14** and **15**.

Reviewers' comments:

Reviewer #2 (Remarks to the Author):

Most of my queries and comments have been addressed in the revised manuscript. However, whether the biology demonstrated in cell lines is also seen in human samples remains unclear, thereby significantly reducing the potential applicability of PIN1 inhibition as a treatment for AR-dependent prostate cancer. I would strongly suggest to validate the differential expression of PIN1 between AR-dependent and independent human prostate cancer samples. Simply stating that " PIN1 is commonly over-expressed in prostate cancer and its expression in patient biopsies correlates with recurrence." does not address the issue.

Reviewers' comments:

Reviewer #2 (Remarks to the Author):
Most of my queries and comments have been addressed in the revised manuscript. However, whether the biology demonstrated in cell lines is also seen in human samples remains unclear, thereby significantly reducing the potential applicability of PIN1 inhibition as a treatment for AR-dependent prostate cancer. I would strongly suggest to validate the differential expression of PIN1 between AR-dependent and independent human prostate cancer samples. Simply stating that " PIN1 is commonly over-expressed in prostate cancer and its expression in patient biopsies correlates with recurrence." does not address the issue.

Response:

As suggested we have included new data that examines levels of Pin1 in AR-dependent and independent human prostate cancer samples (see Supplementary Figure 1 and pages 4-5 in the revised manuscript).

We must reiterate that this study is to examine the potential of a combination treatment that targets both AR and Pin1 for CRPC (adenocarcinoma and not neuroendocrine). This treatment would not be relevant to patients that do not express both of these targets. Neuroendocrine prostate cancer is not treated with AR inhibitors because it is not driven by AR and tends to have low or no expression of AR. Simply put, neuroendocrine prostate cancer is AR-independent and would never be offered a treatment targeting AR (please refer to NCCN Guidelines; a quote from these guidelines is included below). We are struggling to understand what relevance the levels of Pin1 in neuroendocrine disease would have on whether a combination therapy that targets both AR and Pin1 would be effective in CRPC that expresses both proteins.

This manuscript is focused upon the impact of Pin1 on the transcriptional activity of AR to provide the rationale for a therapy that combines a drug that directly binds AR and a drug that inhibits Pin1 enzymatic activity. This is because both of these approaches block AR transcriptional activity, as we show in this manuscript. AR is a ligand-activated transcription factor and its activity is modulated by coactivators and co-repressors. Our findings provide evidence that Pin1 is a coactivator for AR and that co-targeting Pin1 and AR could be beneficial for treating CRPC. We have included new data examining the relationship between Pin1 and the expression of genes that are well-characterized as being regulated by AR in clinical samples of CRPC (Supplementary Figure 1). These data support the premise of elevated expression of PIN1 being associated with increased AR-regulated gene expression. These data are consistent with previous publications entirely focused on measuring the levels of Pin1 in over 500 clinical samples of prostate cancer. As we previously stated, there is an association of Pin1 with PSA recurrence which is an AR-regulated gene (Ayala et al. 2003; Sasaki et al. 2006). In other words, those previous publications reveal levels of PIN1 expression are associated with AR transcriptional activity in clinical studies using PSA as a readout of AR transcriptional activity. Ayala et al. state that “Pin1 expression had a significantly higher probability of recurrence than their counterparts with low Pin1 expression, as defined by a serum prostate-specific antigen level of ≥0.4 ng/ml on two consecutive occasions after radical prostatectomy. In addition, patients with high Pin1 expression had almost 4 times the risk of having earlier recurrence than those

with low Pin1 expression; patients with a very high level had 8.1 times the risk of an earlier recurrence than a low Pin1 expresser." Sasaki et al. reported that "PCa patients assigned to the high Pin1 score group demonstrated PSA relapse more frequently than those assigned to the low Pin1 score group ($p<0.0001$)." These publications describe the relationship between elevated Pin1 expression and PSA relapse which indicates reactivation of the AR transcriptional program. These previous clinical data support the relevance of targeting both AR and Pin1 in CRPC (adenocarcinoma) as we have stated previously and again here.

According to NCCN Guidelines Version 2.2020 Prostate Cancer:

> "Small cell/neuroendocrine carcinoma of the prostate should be considered in patients who no longer respond to ADT and test positive for metastases. These relatively rare tumors are associated with low PSA levels despite large metastatic burden and visceral disease….. These cases may be managed by cytotoxic chemotherapy (ie, cisplatin/etoposide, carboplatin/etoposide, docetaxel/carboplatin). Participation in a clinical trial is another option. Physicians should consult the NCCN Guidelines for Small Cell Lung Cancer (available at www.NCCN.org), because the behavior of small cell/neuroendocrine carcinoma of the prostate is similar to that of small cell carcinoma of the lung." (p108)

ADT: androgen-deprivation therapy, a method of blocking AR transcriptional activity

Also relevant is PROS-15 on page 25 of this guideline that shows the different treatments for CRPC adenocarcinomas versus CRPC neuroendocrine disease. CRPC neuroendocrine disease is treated completely differently and NOT with antagonists of AR transcriptional activity.

REVIEWERS' COMMENTS:

Reviewer #2 (Remarks to the Author):

The authors have satisfactorily addressed my concern. I have no further comments or queries.
